# Muconic acid production from glucose and xylose in *Pseudomonas putida* via evolution and metabolic engineering

**DOI:** 10.1038/s41467-022-32296-y

**Published:** 2022-08-22

**Authors:** Chen Ling, George L. Peabody, Davinia Salvachúa, Young-Mo Kim, Colin M. Kneucker, Christopher H. Calvey, Michela A. Monninger, Nathalie Munoz Munoz, Brenton C. Poirier, Kelsey J. Ramirez, Peter C. St. John, Sean P. Woodworth, Jon K. Magnuson, Kristin E. Burnum-Johnson, Adam M. Guss, Christopher W. Johnson, Gregg T. Beckham

**Affiliations:** 1 Renewable Resources and Enabling Sciences Center, National Renewable Energy Laboratory, Golden, CO 80401 USA; 2 Agile BioFoundry, Emeryville, CA 94608 USA; 3 Biosciences Division, Oak Ridge National Laboratory, Oak Ridge, TN 37831 USA; 4 Pacific Northwest National Laboratory, Richland, WA 99352 USA

**Keywords:** Metabolic engineering, Metabolic engineering, Applied microbiology

## Abstract

Muconic acid is a bioprivileged molecule that can be converted into direct replacement chemicals for incumbent petrochemicals and performance-advantaged bioproducts. In this study, *Pseudomonas putida* KT2440 is engineered to convert glucose and xylose, the primary carbohydrates in lignocellulosic hydrolysates, to muconic acid using a model-guided strategy to maximize the theoretical yield. Using adaptive laboratory evolution (ALE) and metabolic engineering in a strain engineered to express the D-xylose isomerase pathway, we demonstrate that mutations in the heterologous D-xylose:H^+^ symporter (XylE), increased expression of a major facilitator superfamily transporter (PP_2569), and overexpression of *aroB* encoding the native 3-dehydroquinate synthase, enable efficient muconic acid production from glucose and xylose simultaneously. Using the rationally engineered strain, we produce 33.7 g L^−1^ muconate at 0.18 g L^−1^ h^−1^ and a 46% molar yield (92% of the maximum theoretical yield). This engineering strategy is promising for the production of other shikimate pathway-derived compounds from lignocellulosic sugars.

## Abstract (web summary)

Muconic acid is a platform chemical with wide industrial applicability. Here, the authors report efficient muconate production from glucose and xylose by engineered *Pseudomonas putida* strain using adaptive laboratory evolution, metabolic modeling, and rational strain engineering strategies.

## Introduction

The development of economic processes for the production of biofuels and biochemicals from lignocellulose will be critical to help reduce anthropogenic greenhouse gas emissions associated with fossil fuel consumption^1,2^. Among the various areas of metabolic space that have been explored for biochemical production, molecules from microbial aromatic catabolic pathways exhibit substantial chemical diversity^3,4^. Of note, *cis*,*cis*-muconic acid (hereafter muconate) is a popular platform chemical from the catechol catabolic pathway that can be produced from lignin-derived aromatic compounds, carbohydrates and waste plastics-derived aromatic compounds^5–12^. Muconate is a bioprivileged molecule^13^ that can be converted into either direct replacement chemicals, such as adipic acid and terephthalic acid^5,14,15^, or converted to performance-advantaged bioproducts^16–24^.

**Fig. 1:**
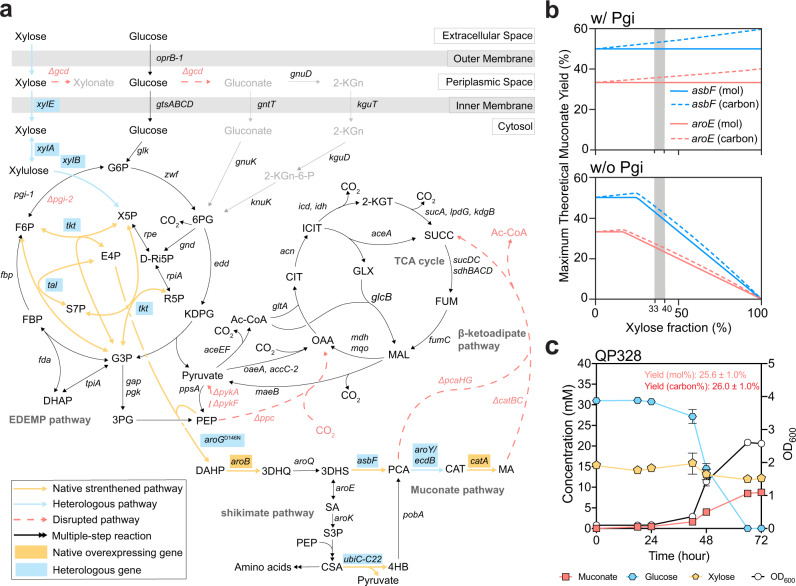
Muconate production from glucose and xylose. **a** Schematic of the overall metabolic engineering strategy. To utilize xylose, *xylE*, d-xylose isomerase (*xylA*), xylulokinase (*xylB*), ﻿transaldolase (*tal*), and transketolase (*tkt*) from *E. coli* were heterologously expressed. E4P and PEP were condensed to form DAHP via a feedback-resistant DAHP synthase (*aroG*^D146N^)^70^. To convert DAHP to muconate (MA), genes encoding a ﻿3-DHS dehydratase (*asbF*) from *Bacillus cereus*, and a PCA decarboxylase (*aroY*) and its corresponding co-factor generating protein (*ecdB*), both from *Enterobacter cloacae*, were heterologously expressed. *aroB* and catechol 1,2-dioxygenase (*catA*) were overexpressed^3,32^. An engineered chorismate pyruvate-lyase from *E. coli* (*ubiC*-C22)^40^ was overexpressed to convert chorismate (CSA) to 4-hydroxybenzoate (4HB), which can be converted to PCA and MA. Deleted genes are shown in red. ﻿Glucose dehydrogenase (*gcd*) was deleted to prevent the formation of xylonate or gluconate. Glucose-6-phosphate isomerases *pgi-1* and *pgi-2* were each deleted previously^3,32^, but *pgi-1* was restored in this study. Pyruvate kinases *pykA* and *pykF* were each deleted to reduce competition for PEP. To accumulate MA, *pcaHG* and *catBC* were deleted to prevent ring-opening of PCA and catabolism of MA, respectively. P phosphate, 2-KGn 2-ketogluconate, 2-KG-6-P 2-ketogluconate-6-P, G6P glucose-6-P, 6PG 6-phosphogluconate, KDPG 2-keto-3-deoxy-6-phosphogluconate, G3P glyceraldehyde-3-P, FBP fructose-1,6-P_2_, F6P fructose-6-P, S7P sedoheptulose-7-P, R5P ribose-5-P, Ri5P ribulose-5-P, 3PG 3-phosphoglycerate, CAT catechol, SA shikimate, S3P shikimate-3-phosphate, ICIT isocitrate, CIT citrate, AKG alpha-ketoglutarate, SUCC succinate, FUM fumarate, MAL malate, GLX glyoxylate, OAA oxaloacetate, AcCoA acetyl-Coenzyme A. **b** Metabolic modeling of the maximum theoretical muconate molar and carbon yields with or without *pgi-1*. The blue lines represent the *asbF* pathway, the red lines represent the *aroE* pathway, solid lines represent % molar yield, and dashed lines represent % carbon yield. The gray areas represent the molar percentage of xylose consumed from 33 to 40% (with the balance glucose), mimicking the composition of corn stover hydrolysates. **c** Shake-flask cultivations of strain QP328 on glucose and xylose. % Molar yield was calculated as [mM muconate/mM (glucose + xylose) × 100], and % carbon yield was calculated as [mM muconate × 6/mM (glucose × 6 + xylose × 5) × 100]. Error bars represent the standard deviation of biological triplicates. Source data are provided as a Source Data file.

Muconate production from carbohydrates is based on the shikimate pathway for aromatic amino acid biosynthesis and was first demonstrated in recombinant *Escherichia coli*^5^. Erythrose-4-phosphate (E4P) and phosphoenolpyruvate (PEP) are condensed to form 3-deoxy-d-arabinoheptulosonate 7-phosphate (DAHP), which is further converted to 3-dehydroshikimate (3-DHS), a key intermediate in the shikimate pathway. From 3-DHS, at least five pathways have been reported for muconate biosynthesis^25–30^. Among these pathways, one ﻿proceeds through the intermediate protocatechuate (PCA) via a 3-DHS dehydratase (*asbF*) and results in a higher maximum theoretical yield than the others, which proceed through shikimate via a shikimate dehydrogenase (*aroE*) (Fig. 1a)^29^.

Several previous efforts to produce muconate from sugars via *asbF* have disrupted the shikimate pathway by deleting *aroE*^10,11,31^. Deletion of *aroE* results in strains that are auxotrophic for essential aromatic amino acids, which is undesirable for a bioprocess^10,25^. Recently, *Pseudomonas putida* KT2440 (hereafter *P. putida*) strains have been engineered to efficiently produce muconate from glucose via *asbF*^3,32,33^. Most recently, we reported the engineering of *P. putida* that achieved a titer of 22.0 g L^−1^ at 0.21 g L^−1^ h^−1^ and a 35.6% molar yield from glucose in a pH-controlled bioreactor^32^.

To date, most efforts to produce muconate from carbohydrates have employed glucose as a substrate. However, the co-utilization of glucose and xylose—often are the two major carbohydrates in lignocellulose^2^— is crucial for the valorization of biomass hydrolysates. Co-utilization of glucose and xylose for muconate production has been studied in ﻿*Escherichia coli*^11^. In this previous work, xylose was metabolized to the TCA cycle to avoid ﻿carbon catabolite repression (CCR), thus limiting muconate yield, which motivates the development of other strategies toward this goal. Unlike *E. coli*, *P. putida* is natively unable to utilize xylose, which provides an opportunity to engineer optimal xylose pathways in the absence of CCR^34–37^.

In this work, ﻿we seek to incorporate xylose utilization to achieve efficient muconate production from glucose and xylose in *P. putida*. To this end, we first delete *hexR* and engineer the D-xylose isomerase pathway into a strain previously engineered to produce muconate from glucose (Table 1). By combining metabolic modeling, rational strain engineering, adaptive laboratory evolution, and bioreactors cultivation, we identify successful strategies to improve muconate production from glucose and xylose. Finally, metabolomics is performed to infer the impact of the genetic modifications on metabolic flux.

**Table 1:** Strains in this study

Strain	Genotype	References
CJ522	*P. putida* KT2440 Δ*catRBC*::P*tac*:*catA* Δ*pcaHG*::P*tac*:*aroY*:*ecdB*:*asbF* Δ*pykA*::*aroG-D146N*:*aroY*:*ecdB*:*asbF* Δ*pykF* Δ*ppc* Δ*pgi-1* Δ*pgi-2* Δ*gcd*	3
JE3226	*P. putida* KT2440 Δ*hsdR*::P_*tac*_:BxB1*int*-*attB* Δ*gcd* Δ*ampC*::P_xylE*_xylE:*xylAB*:*tktA*:*talB*	35
JE3692	JE3226 ∆*gcd::araE-araCDABE*	35
QP328	CJ522 Δ*hexR* Δ*ampC*::P_xylE*_xylE:P*tac*:*xylAB*:*talB*:*tktA* ∆*pgi-1*::*pgi-1* PP_1736-1737(intergenic)::Plac:*ubiC*-C22	This study
QP478	QP328 *xylE-A62V*, *A455V* P_PP_2569_ G→A duplication of PP_5050–PP_5242	This study
LC041	JE3226 ∆*pgi-1*	This study
LC061	QP478 restoration of G→A at P_PP_2569_	This study
LC078	QP478 restoration of *xylE-A455V*	This study
LC093	QP478 restoration of *xylE-A62V*	This study
LC091	QP328 *xylE-A62V, A455V*	This study
LC092	QP328 P_PP_2569_ G→A	This study
LC100	LC091 P_PP_2569_ G→A	This study
LC111	JE3692 *xylE-A62V*, *A455V*	This study
LC147	LC100 Δ*pykF*::P_*lac*_:*gpmI*	This study
LC150	LC100 Δ*pykF*::P_*tac*_:*maeB*	This study
LC151	LC100 Δ*pykF*::P_*tac*_:*rpiA*	This study
LC168	LC100 Δ*pykF*::P_*tac*_:*aroK*:*aroB*	This study
LC171	QP478 ΔPP_5050–PP_5242	This study
LC173	QP478 ΔPP_5084–PP_5242	This study
LC199	LC100 Δ*pykF*::P_*tac*_:*aroK*	This study
LC224	LC100 Δ*pykF*::P_*tac*_:*aroB*	This study
LC345	JE3226 ∆*pgi-2*	This study
LC347	LC041 ∆*pgi-2*	This study
LC349	QP328 Δ*pykF*::P_*tac*_:*aroB*	This study

^*^Represents mutation in promoter of xylE^35^.

## Results

### Introducing the d-xylose isomerase pathway into muconate-producing *P. putida*

Three xylose metabolic pathways were considered to enable the production of muconate from this substrate^36^, including the isomerase pathway in which xylose is metabolized to ﻿xylulose-5-P (X5P) in the pentose phosphate pathway (PPP)^38^, the Weimberg pathway that feeds xylose to the TCA cycle via α-ketoglutarate^38,39^, and the Dahms pathway^40^, which shares the initial three steps with the Weimberg pathway, after which ﻿α-ketoglutaric semialdehyde is converted by an aldolase into pyruvate and glycolaldehyde. Among these, the d-xylose isomerase pathway, in which xylose is metabolized via the ﻿d-xylose isomerase (*xylA*) and xylulokinase (*xylB*) to ﻿xylulose-5-phosphate (X5P), is ideal for achieving a high theoretical muconate yield since X5P can be further converted to E4P and subsequently enter the shikimate pathway (Fig. 1a)^35^. We integrated the isomerase pathway into a strain previously engineered to produce muconate from glucose, CJ522^3^, by overexpressing ﻿codon-optimized versions of the *E. coli* d-xylose isomerase (*xylA*), xylulokinase (*xylB*), and d-xylose:H^+^ symporter (*xylE*), together with a transaldolase (*tal*) and a transketolase (*tkt*) to improve carbon flux within the PPP (Fig. 1a)^35^. We also deleted *hexR*, which encodes a transcriptional regulator that controls expression of genes important for sugar metabolism, since we had previously found this to improve the conversion of glucose to muconate^32^.

Thompson et al. previously reported that employing both the *asbF* and *aroE* pathways can help to ﻿maximize net precursor assimilation and metabolite flux toward muconate^25^. Thus, an engineered ﻿chorismate pyruvate-lyase (*ubiC-C22*)^41^ ﻿with relieved product inhibition was integrated to enhance muconate production through the shikimate pathway via *aroE* (Fig. 1a). We had previously deleted *pgi-1* and *pgi-2*, which encode redundant glucose-6-P isomerases, to disrupt the EDEMP cycle, a combination of the Entner-Doudoroff, gluconeogenic Embden-Meyerhoff-Pernass, and the pentose phosphate pathways^42^. The purpose of disrupting the EDEMP cycle is to prevent it from cycling to generate pyruvate independent of PEP during growth on glucose, which could enable the cell to redirect carbon toward growth at the expense of muconate production, despite deletion of the genes encoding the pyruvate kinases (*pykA*, *pykF*) and PEP carboxylase (*ppc*)^3^. This strategy is beneficial for muconate production from glucose as the sole carbon source, but in this case, deletion of *pgi-1* and *pgi-2* would decrease the maximum theoretical muconate yield of both *asbF-* and *aroE-*catalyzed muconate biosynthesis pathways when xylose is converted via the PPP (Fig. 1b).

Considering that the xylose fraction in the mixture of glucose and xylose (xylose/glucose+xylose%, moles) in corn stover hydrolysate ranges from 34 to 38% (Supplementary Fig. 1), the modeling predicted maximum theoretical yield of muconate with *pgi-1* and *pgi-2* deleted to be lower than if one or both are present (Fig. 1b). To test the hypothesis that glucose-6-phosphate isomerase (encoded by *pgi-1* and *pgi-2*) activity is necessary for xylose flux to enter the EDEMP cycle, we built strains based on JE3226^35^, a *P. putida* KT2440-dervied strain that was previously engineered to utilize xylose using the d-xylose isomerase pathway but is otherwise wild-type, generating strains LC041 (∆*pgi-1*), LC345 (∆*pgi-2*), LC347 (∆*pgi-1* ∆*pgi-2*). In plate reader cultivation on M9 medium containing xylose, LC347 failed to grow, whereas both LC041 and LC345 demonstrated reduced growth rates and increased growth lags (Supplementary Fig. 2). LC345, with *pgi-1* intact, exhibited a lower growth rate and longer growth lag compared to LC041, which contains only *pgi-2*, suggesting that Pgi-1 contributes less to the overall glucose-6-phosphate isomerase activity than Pgi-2. Since the EDEMP cycle would be expected to compete with muconate biosynthesis and reduce the muconate yield, we thus restored *pgi-1* to enable xylose flux into the EDEMP cycle and improve the maximum theoretical yield, generating strain QP328 (Fig. 1a and Table 1).

Strain QP328 was cultivated in shake flasks on a mixture of glucose and xylose to examine their conversion to muconate. Although the xylose isomerase pathway has been shown to be efficient in wild-type *P. putida*^35^, the xylose utilization rate of QP328, however, was very low compared to that of glucose (Fig. 1c). Since glucose and xylose can be utilized simultaneously in the *P. putida* KT2440 wild-type background upon introduction of the same xylose isomerase pathway^35^, we hypothesized that a bottleneck in xylose transport or metabolism was present in our muconate-producing strain.

### ALE of QP328 to improve growth on xylose

**Fig. 2:**
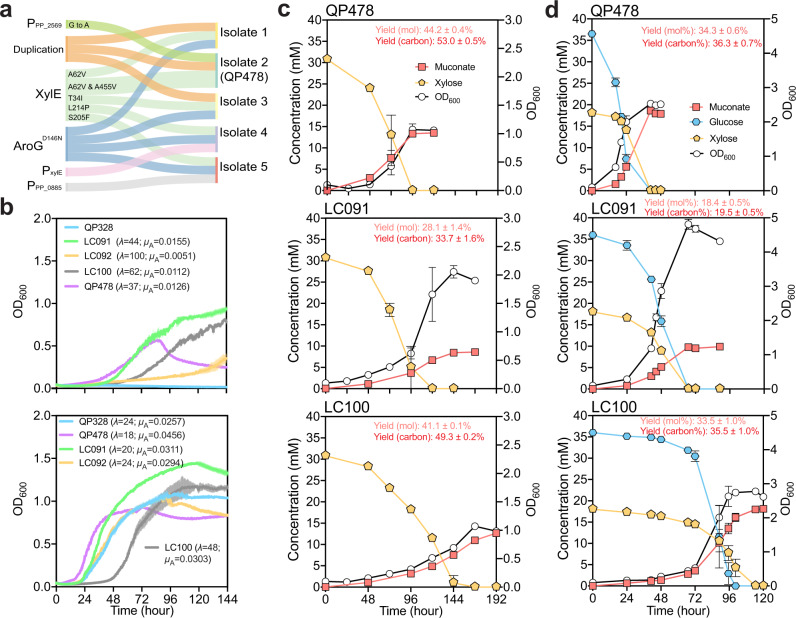
Evaluation of reverse engineered strains comparing to QP478. **a** Mutations identified in ALE by whole-genome sequencing of the five isolates (the Sankey diagram was built using SankeyMATIC online tool). **b** Growth curve of reverse engineered strains LC091 and LC100, with the unevolved strain QP328 and the evolved strain QP478 for comparison. *μ*_A_ represents absolute growth rate, and all *μ*_A_ presented here are the average values of three independent growth curves. **c** Shake-flask experiments of reverse engineered strains LC091 and LC100, comparing to QP478, on M9 medium supplemented with 30 mM xylose. Yield of LC100 was compared to LC091 using two-tailed Student *t* test (*P* < 0.0001). **d** Shake flask experiments comparing the reverse engineered strains LC091 and LC100, and the evolved strain QP478 on M9 medium supplemented with 30 mM glucose and 15 mM xylose. % Molar yield was calculated as [mM muconate/mM (glucose + xylose) × 100], and % carbon yield was calculated as [mM muconate × 6/mM (glucose × 6 + xylose × 5) × 100]. Error bars here represent the standard deviation of three biological replicates. Source data are provided as a Source Data file.

To improve xylose utilization by QP328, ﻿we conducted ALE by serial passaging of the strain on M9 medium supplemented with 10 mM xylose as a sole carbon and energy source. As the populations were passaged, higher OD_600_ values were achieved more rapidly. After 7 passages (﻿~50 generations), all four lineages achieved turbidity in 2–4 days compared to 14 days at the beginning of the ALE, and the evolution was terminated. The evolved populations of the four lineages were plated onto an LB agar plate, and three isolates on each plate were chosen for shake flask pre-screening (12 isolates in total). In most cases, all triplicates from the same lineage exhibited similar growth and muconate production, so it was assumed that they likely represented the same genotype and only one from each lineage was saved. In lineage 1, however, one replicate performed differently, thus two isolates were saved (Supplementary Fig. 3). To identify mutations that may contribute to improved xylose utilization, the genomes of all five isolates were sequenced. All five isolates had mutations in xylose transporter *xylE* (A62V, A62V and A455V, T34I, L214P, S205F, for isolates 1, 2, 3, 4, 5, respectively). Four of the isolates (1, 3–5) had mutations that likely inactivated *aroG-D146N* (frameshift +7 bp, frameshift +2 bp, M1N and L2H, frameshift −16 bp, for isolate 1, 3, 4, 5, respectively) (Fig. 2a). The five isolates were then evaluated in shake flasks on glucose, xylose, and a mixture of glucose and xylose. As expected, strains with mutations in *aroG-D146N* grew better but produced less muconate. Isolate 2 achieved the highest muconate yield and the lowest biomass yield, and was designated QP478 (Supplementary Fig. 4). QP478 demonstrated substantially improved growth on xylose compared to QP328 in a plate reader, in which the growth of QP328 was negligible while QP478 reached a OD_600_ of 0.5 in 72 h (Fig. 2b).

The mutations identified in QP478 are listed in Supplementary Data 1. Of those, mutations we hypothesized might be related to the improved growth on xylose included: (1) two missense mutations in the xylose transporter gene, *xylE*, where alanine residues were replaced with valines, A62V and A455V; (2) a G→A point mutation 10 bp upstream of the −35 element of a putative promoter (Supplementary Fig. 5) predicted ﻿by the BPROM σ70 promoter prediction program^43^ upstream of PP_2569, which is annotated as a metabolite major facilitator superfamily (MFS) transporter in the Uniprot database; and (3) a 227.8 kB region of the genome from PP_5050 to PP_5242 that appeared to be duplicated (Fig. 2a).

### Evaluation and reverse engineering of the ALE-derived mutations

To understand the contribution of the mutations that led to improved growth on xylose during ALE, we created strains that individually restored the wild-type sequences into the evolved strain QP478. The A62V and A455V mutations were restored to wild type separately in *xylE*, generating LC093 and LC078, respectively. The G→A mutation in the promoter region of PP_2569 was restored, generating LC061. In plate reader experiments, restoring either *xylE-A455V* or *xylE-A62V* led to decreased growth rate and increased growth lag of LC078 and LC093, respectively (Supplementary Fig. 6a–c). The restoration of the G→A mutation in P_PP_2569_ led to slightly decreased growth rate (Supplementary Fig. 6a, b). In shake flasks, only LC093 demonstrated significantly lower muconate yield compared to QP478 (Supplementary Fig. 6f, g, j), but all three strains LC061, LC078, and LC093 exhibited slower growth and muconate production (Supplementary Fig. 6g–j), which is consistent with the results from the plate reader experiments (Supplementary Fig. 6a–c). The reduced muconate productivity, caused by decreased growth rates and/or increased growth lag, indicated that all these mutations contributed to improved cell growth on xylose of QP478 (Supplementary Fig. 6).

We also performed the reverse experiment, engineering the ALE mutations into the parent strain QP328 to obtain a rationally engineered strain containing only mutations that contribute to improved production of muconate. We first reverse engineered the unevolved strain QP328 with the three point mutations. The A62V and A455V XylE mutations were introduced into the unevolved strain QP328, generating LC091. The G→A mutation in P_PP_2569_ was engineered in QP328 and LC091, generating LC092 and LC100, respectively. Strains LC091, LC092, and LC100, together with QP328 and QP478, were evaluated in a plate reader containing M9 medium with 30 mM xylose. Interestingly, introducing the two XylE mutations enabled cell growth on xylose in LC091 (Fig. 2b), which exhibited a comparable growth rate but higher final biomass compared to QP478 on xylose alone and xylose and glucose mixture (Fig. 2b). Introducing the G→A mutation in P_PP_2569_ to QP328 also enabled cell growth of LC092 on xylose, while at a much lower rate compared to LC091 (Fig. 2b). Unexpectedly, introducing the G→A mutation in P_PP_2569_ to LC091 led to decreased growth and lower biomass of LC100 on both xylose and mixed substrates (Fig. 2b).

We next evaluated LC091, LC100, and QP478 in shake flasks containing M9 medium with 30 mM xylose. LC091 reached almost twice the biomass yield (OD_600_) but achieved lower muconate yield compared to QP478 (Fig. 2c). Moreover, LC100 reached a comparable muconate yield to QP478, albeit at a lower rate (Fig. 2c). RT-qPCR indicated that the G→A mutation in P_PP_2569_ increased the expression of PP_2569 (Supplementary Fig. 7). We also evaluated LC091, LC100, and QP478 on M9 medium with a mixture of glucose and xylose. Consistent with the results on xylose only, the muconate yield of LC091 is lower than LC100 and QP478 on the mixture; LC100 still exhibited much slower growth than QP478 on the mixture, though it utilized glucose and xylose simultaneously and reached comparable muconate yield (Fig. 2d). The difference in these strains suggested that a gene or genes in the PP_5050–PP_5242 duplicated region might be important for the improved performance of QP478.

### Investigation into mechanisms of the mutations

**Fig. 3:**
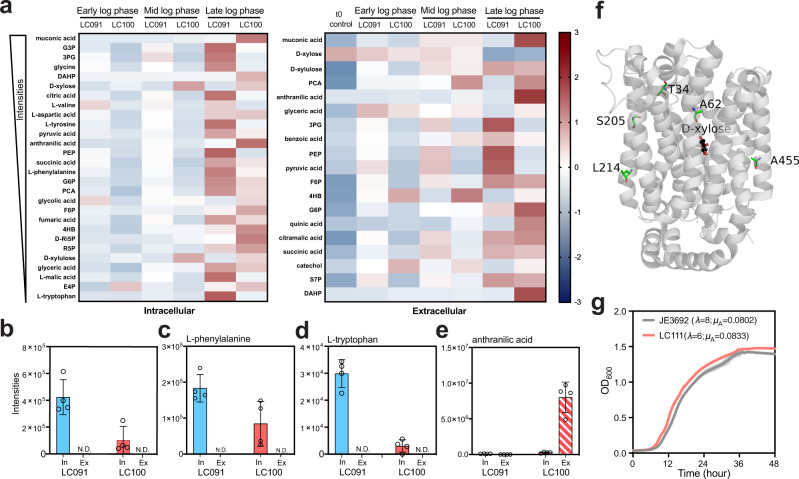
Characterizing mutations in the promoter region of PP_2569 and XylE. **a** Heatmap of the selected intracellular and extracellular metabolites. Z-scores were calculated using the average intensities of intracellular and extracellular metabolites separately, and a time zero control from uninoculated medium was also included for extracellular metabolite analysis. **b**–**e** Intensities of selected metabolites from late log phase, “In” represents intracellular, “Ex” represents extracellular, and “N.D.” represents not detected. The intracellular intensity signal was collected using the cell pellet from a 1 mL cell culture, and the extracellular intensity signal was collected using 20 μL of the corresponding supernatant. **f** Structure of XylE (PDB ID: 4GBY)^48^ with residues of ALE-derived mutation locations labeled in green. d-xylose is shown in black ball-and-sticks. **g** Growth curve of strain LC111 compared to JE3692 on M9 medium supplemented with 30 mM xylose. Source data are provided as a Source Data file.

The only genetic difference between strains LC091 and LC100 is the G→A mutation in the promoter region that led to higher expression of a putative MFS transporter PP_2569 (Supplementary Fig. 5). To rationalize how the mutation could occur in ALE, and to examine how metabolism could be affected by PP_2569, intracellular and extracellular metabolomics experiments were conducted with LC091 and LC100 grown on xylose. Selected metabolites from early log phase, mid-log phase, and late log phase were analyzed (Supplementary Fig. 8), and Z-scores were plotted as Fig. 3a. Generally, we observed that LC091 accumulated more metabolites in both the EDEMP pathway and the TCA cycle, both intracellularly and extracellularly, while LC100 demonstrated a greater accumulation of shikimate pathway-related metabolites (Fig. 3a). There were three prominent exceptions of shikimate pathway-related compounds that were more abundant in LC091, namely l-tyrosine, l-phenylalanine, and l-tryptophan, which are all chorismate-derived aromatic amino acids (Fig. 3b–d). In *Pseudomonas aeruginosa*, it was previously reported that l-tyrosine and l-tryptophan could strongly inhibit the native DAHP synthases AroF-1 and AroF-2^44^. Thus, we posit that AroF-1 and AroF-2 could be less inhibited in LC100 compared to LC091. Although a feedback-resistant DAHP synthase *aroG*^*D146N*^ has been overexpressed in our strains, previous studies showed that the native DAHP synthases AroF-1 and AroF-2 alone led to ~30% PCA and phenol production compared to additional overexpression of *aroG*^*D146N*^, in *P. putida*^45^ and *Pseudomonas taiwanensis*^46^, respectively. We reasoned that the improved muconate production of LC100 compared to LC091 might be because of lower inhibition of AroF-1 and AroF-2 (Fig. 3b–e), and the reduced PEP concentration and increased accumulation of DAHP in LC100 support this interpretation (Fig. 3a).

Moreover, a further explanation could be that PP_2569 is able to transport the aromatic amino acids extracellularly—however, we did not observe the extracellular accumulation of these amino acids (Fig. 3b–d). Instead, we found substantial extracellular accumulation of anthranilic acid in LC100 (Fig. 3e). Anthranilic acid is a precursor of l-tryptophan and direct product of chorismate, which is an important node in the shikimate pathway from which all of the aromatic amino acids are derived. Chorismate was reported to be unstable intracellularly^47^ and was not detected in our intracellular metabolomics samples. Based on the current results, we posited that PP_2569 might be able to export anthranilic acid, which could lead to decreased l-tryptophan intracellular accumulation (Fig. 3d, e). The increased accumulation of anthranilic acid may also reduce the flux from chorismate toward other aromatic amino acids, leading to the reduced accumulation of l-tyrosine and l-phenylalanine in strain LC100. Further work is warranted to investigate the mechanistic basis of this beneficial ALE-derived mutation.

Separately, to investigate the mechanism of mutations in XylE, five mutations from isolates 1–5 (A62V, A62V, and A455V, T34I, L214P, S205F, for isolates 1, 2, 3, 4, 5, respectively) were labeled in the structure (PDB ID: 4GBY)^48^, highlighting that the mutations were all located in the transmembrane domains (Fig. 3f). Previously, Jiang et al. demonstrated that introducing two mutations (G58W and L315W) in XylE could prevent the binding of two inhibitors through conformational changes^49^. Notably, the G58W site is in the same transmembrane domain to A62V and close to T34I in the structure. Since the non-muconate-producing strain LC345 with wild-type XylE and similar genetic background to QP328 grew well on xylose (Supplementary Fig. 2a), we hypothesized that the mutations in XylE that occurred in ALE might be induced by inhibitor(s) from the muconate-producing background strain. We also introduced the mutations *xylE*-*A62V* and *A455V* to strain JE3692, previously reported to grow on lignocellulosic hydrolysates, generating LC111. The two strains were evaluated in a plate reader on xylose. LC111 demonstrated improved growth with a reduced lag time and higher growth rate compared to JE3692 (Fig. 3g). The slight improvement may be caused by the trace amounts of inhibitor(s) from the native pathways, and this may also suggest that introducing *xylE*-*A62V*, *A455V* can improve xylose utilization for the production of other non-shikimate pathway-related products.

### Investigation of the PP_5050–PP_5242 duplication

**Fig. 4:**
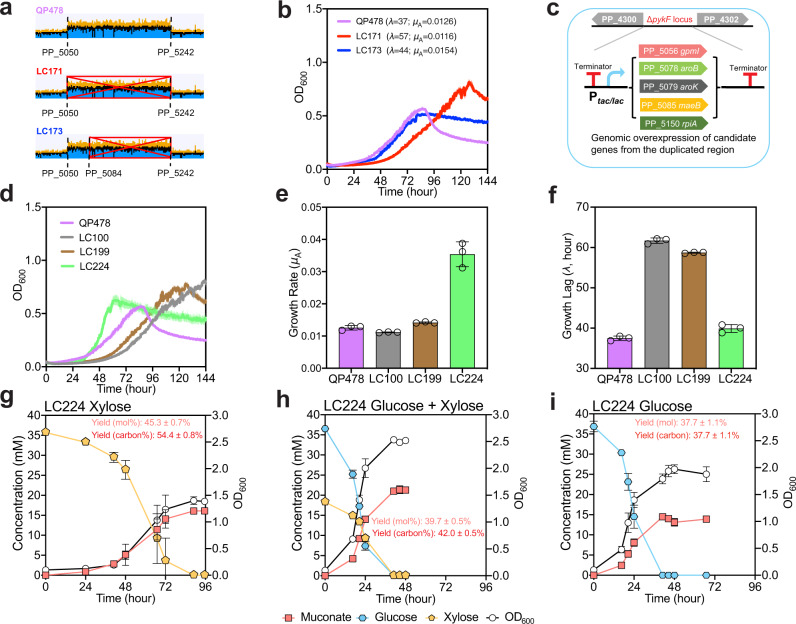
Characterization of rationally engineered strains overexpressing genes from the duplicated region. **a** Identification of the duplicated region from next-generation sequencing data, which presented ~2× the number of sequencing reads in QP478, and complete and partial deletions of these duplicated regions in LC171 and LC173, respectively. The graphs of coverage were generated in Geneious Prime 2020.0.4. **b** Growth curves of QP478, LC171, and LC173 on M9 medium with 30 mM xylose. *λ* represents growth lag, *μ*_A_ represents absolute growth rate, and both are the average values of three independent growth curves. **c** Overexpressing candidate genes in reverse engineered strain LC100 at the Δ*pykF* site. **d** Growth curves of QP478, LC100, LC199 and LC224 on M9 medium with 30 mM xylose. **e** Maximum specific growth rates extracted from panel **d**. **f** Growth lag values extracted from panel **d**. **g**–**i** Profiles in shake-flask experiments of strain LC224 on M9 medium with 30 mM xylose, 30 mM glucose + 15 mM xylose, and 30 mM glucose, respectively. For shake flask experiments, % molar yield was calculated as [mM muconate/mM (glucose + xylose) × 100], and % carbon yield was calculated as [mM muconate × 6/mM (glucose × 6 + xylose × 5) × 100]. Error bars here represent the standard deviation of three biological replicates. Source data are provided as a Source Data file.

The 227.8 kB duplication was identified based on approximately twofold higher sequencing coverage from PP_5050 to PP_5242 compared to the rest of the genome (Fig. 4a). However, it was challenging to identify the exact location of the duplicated region based on the sequencing data due to the short read length of Illumina sequencing^50,51^. We thus deleted the original region of PP_5050–PP_5242 in QP478, using the known sequences outside the region as homologous arms, to generate LC171, and deleted a portion of the duplication from PP_5084–PP_5242 to generate strain LC173. On 30 mM xylose in M9 medium, LC171 with the whole region deletion exhibited a lower growth rate, while LC173 with partial deletion showed comparable growth with a slightly longer growth lag and increased growth rate relative to QP478 (Fig. 4b). Based on these results, we concluded that the duplication is important to the performance of QP478, and the potential beneficial gene(s) should be in the region PP_5050–PP_5083, which remains intact in LC173. Thus, we next sought to identify the gene(s) in this region that contributed to the improved growth on xylose.

Since glucose and xylose were both utilized at similarly low rates in LC100 (Fig. 2d), we reasoned that the slow growth might manifest in part(s) of the pathway shared by both sugars. Three candidate genes within PP_5050–PP_5083 were selected for overexpression in LC100, including one related with sugar metabolism, PP_5056 (*gpmI*, 2,3-bisphosphoglycerate-independent phosphoglycerate mutase), and two in the shikimate pathway, PP_5078 (*aroB*, 3-dehydroquinate synthase) and PP_5079 (*aroK*, shikimate kinase) (Fig. 4c). Two other genes outside this region but related with sugar metabolism were also tested, including PP_5085 (*maeB*, malic enzyme B) and PP_5150 (*rpiA*, ribose-5-phosphate isomerase A). Overexpression cassettes of the five genes were then inserted individually at the Δ*pykF* site, generating strains LC147 (*gpmI*), LC150 (*maeB*), LC151 (*rpiA*), LC199 (*aroK*), and LC224 (*aroB*). All of these genes were driven by the P_*tac*_ promoter except for *gpmI*, which was driven by P_*lac*_ promoter after two unsuccessful attempts to insert the gene into the genome using P_*tac*_. The resulting strains were then evaluated with LC100 and QP478 in a plate reader containing M9 medium and 30 mM xylose (Fig. 4d–f). Overexpression of the three genes related to sugar metabolism in LC100 did not reduce the growth lag, while strains LC150 and LC151 demonstrated higher growth rates and greater final biomass accumulation (Supplementary Fig. 9). Considering that higher biomass yield may reduce muconate yield, as we observed with strain LC091 (Fig. 2b–d), we decided to not pursue these targets further. Strains LC199 and LC224, which overexpress *aroK* and *aroB*, respectively, both demonstrated improved growth rates and reduced growth lag compared to LC100 (Fig. 4d–f). LC224 grew even faster than QP478 with a similar lag time and higher growth rate (Fig. 4d–f).

To investigate the potential additive effect of overexpressing *aroK* and *aroB*, we also expressed *aroK* and *aroB* in an operon-like pattern as *aroKB* in LC100, generating strain LC168. Strains LC199, LC224, LC168, and QP478 were evaluated in shake-flask experiments with M9 medium containing glucose and xylose to examine muconate production. The *aroB* overexpression strain LC224 outperformed its evolved counterpart QP478 with a higher muconate yield and improved growth rate (Supplementary Fig. 10a, c), suggesting that the reaction of DAHP to 3-dehydroquinate (3DHQ) was rate limiting in LC100. Overexpressing *aroK* in LC100 (generating LC199) increased the growth rate slightly (Supplementary Fig. 10b, d). Strain LC168 did not exhibit improvement compared to LC224 (Supplementary Fig. 10c, e).

To investigate if *aroB* overexpression alone can lead to better strain performance, we overexpressed *aroB* in QP328, generating strain LC349. In the plate reader evaluation of strains LC349, QP328 and LC224, LC349 exhibited highest growth rate on glucose, and slightly lower growth rate on mixture of glucose and xylose compared to LC224, while not surprisingly much slower growth on xylose relative to LC224 (Supplementary Fig. 11a–c), probably due to the lack of mutations in *xylE*. Interestingly, in shake flasks experiment on mixture of glucose and xylose, LC349 outperformed QP328 with a much higher muconate yield, which was still significantly lower than LC224 (Supplementary Fig. 11d–f). The muconate production of LC349 is slower than LC224, as it took up to 41 h for LC349 and 26.5 h for LC224 to reach maximum muconate titer (Supplementary Fig. 11e, f).

We next examined the performance of LC224 on M9 medium containing glucose, xylose, or a mixture of the two. Muconate yields were highest on xylose, lowest on glucose, and intermediate on the mixture (Fig. 4g–i), reflecting the benefit of introducing xylose into the pentose phosphate pathway to supply E4P. Interestingly, both glucose and xylose utilization rates were higher on the mixture of glucose and xylose compared to on glucose or xylose alone (Fig. 4g–i).

### Bioreactor cultivations to assess strain performance

**Fig. 5:**
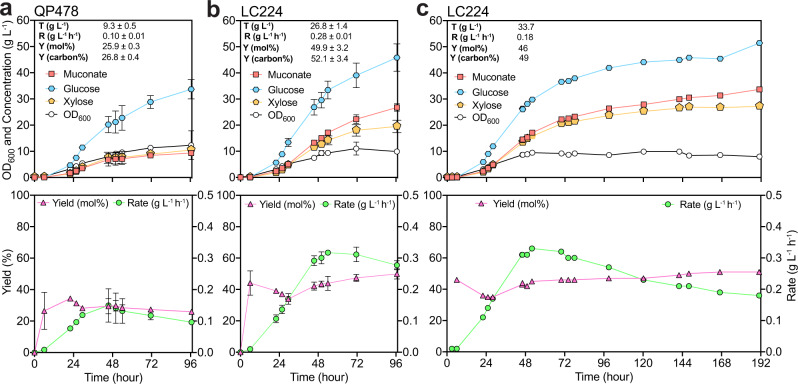
Bioreactor profiles from strains QP478 and LC224 in fed-batch mode. **a**, **b** Bacterial growth, glucose and xylose utilization, and muconate titers, yields, and rates from QP478 and LC224 in 96.6-h cultivations. **c** Bacterial growth, glucose and xylose utilization, and muconate titer, yields, and rate from LC224 in 191-h cultivation. For **a** and **b**, data points represent the average values of biological duplicates, error bars represent the absolute difference between the data generated from duplicates at each time point; for (**c**), data points represent the values of singlets. Metabolic yields (mol%) at each time point were calculated as the amount of muconate (in moles) produced divided by the glucose and xylose (in moles) utilized. Metabolic yields (mol%) are corrected based on the dilution factor generated by the volumes of base and substrate feeding. Final carbon yields (carbon%) listed were calculated as [mM muconate × 6/mM (glucose × 6 + xylose × 5) × 100]. Rates (g L^−1^ h^−1^) at each time point were calculated as the muconate concentration divided by the cultivation time. All the titer (T), rate (R), and yield (Y) values listed were at the last time point. Final yields (mol%, carbon%) listed in (**c**) have also been corrected based on the quantified evaporated volume. Source data are provided as a Source Data file.

Bioreactor cultivations of LC224 and QP478 were conducted in fed-batch mode to maintain sugar (glucose and xylose) concentrations lower than 10 g L^−1^ (Supplementary Fig. 12a–c). Glucose and xylose were simultaneously utilized in both strains from the start of the cultivation (Fig. 5a, b); however, sugar utilization rates were higher in LC224 than QP478. LC224 utilized 46 g L^−1^ glucose and 20 g L^−1^ of xylose by the end of the cultivation while QP478 utilized 34 g L^−1^ and 10 g L^−1^, respectively. The muconate titer was almost threefold higher in LC224 compared to QP478, a 26.8 g L^−1^ and 9.3 g L^−1^, respectively (Fig. 5a, b). Muconate yields reached 50% (molar yield) in LC224 while the yields were 25.9% in QP478 (Fig. 5a, b). These improvements in LC224 were also reflected in the overall muconate production rate (0.28 g L^−1^ h^−1^), which was substantially higher than that achieved in QP478 (0.10 g L^−1^ h^−1^) (Fig. 5a, b).

The muconate titer, rate, and yield achieved in bioreactor cultivations were 26.8 g L^−1^, 0.28 g L^−1^ h^−1^, and 49.9% (Fig. 5b), respectively, at 96.6 h. This yield represents almost 100% of the maximum theoretical based on our strain design and metabolic modeling (vida supra). To explore whether the titer could be further improved, we conducted another bioreactor experiment where LC224 was cultivated for 191 h (Fig. 5c). The resulting muconate titer increased to 33.7 g L^−1^ and at a yield of 46%, 92% of the theoretical maximum when corrected for evaporation. It is also noteworthy that while LC224 reached stationary phase at ~54 h, the cells continued utilizing sugars and producing muconate, which demonstrated that the muconate production here was not growth coupled, suggesting that the muconate titer and yield could be further improved if the experiment had continued (Fig. 5b, c).

### Metabolomic analysis of QP328, QP478, and LC224 cultivated on glucose and xylose

**Fig. 6:**
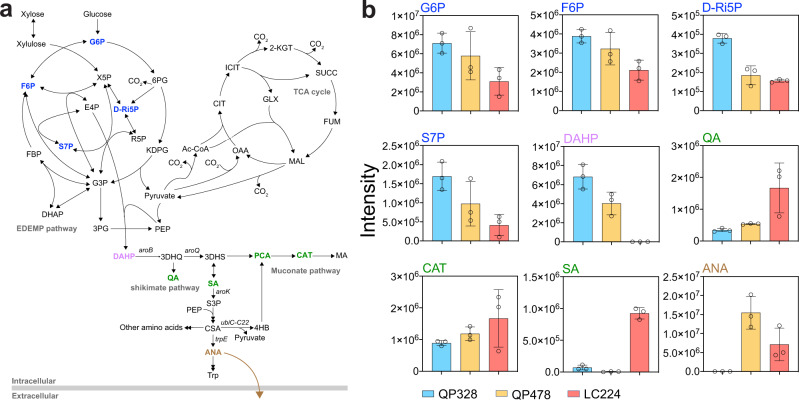
Selected intracellular and extracellular metabolomics analysis of strains grown on glucose and xylose. **a** Simplified metabolic pathway. Metabolites in the EDEMP cycle are labeled blue, in the shikimate and muconate pathway are labeled green, the joint node DAHP is labeled purple, the extracellular anthranilic acid (ANA) is labeled brown. QA quinic acid, ANA anthranilic acid. **b** Intensity of selected metabolites in the three strains QP328 (blue), QP478 (orange), and LC224 (red). Intracellular intensities have been normalized by lyophilized biomass. Error bars represent the standard deviation of three biological replicates. Source data are provided as a Source Data file.

To better understand the differences between the unevolved parent QP328, the evolved strain QP478, and the rationally engineered strain LC224, intracellular and extracellular metabolomics experiments were conducted. Selected metabolites related to sugar metabolism and muconate production are presented in Fig. 6. Compared to QP328, the strains QP478 and LC224 exhibited reduced accumulation of metabolites in the EDEMP cycle, and greater accumulation of metabolites in the shikimate and the muconate pathways (Fig. 6a, b), which is consistent with the mutations that evolved in QP478 and were engineered in LC224 (Figs. 2 and 4). The intensities of DAHP, the joint node of sugar metabolism and shikimate pathway, however, demonstrated the opposite pattern compared to other metabolites in shikimate pathway (Fig. 6b). LC224 and QP478 accumulated less DAHP compared to QP328, which is consistent to our objective above regarding the duplication and *aroB* overexpression.

Specifically, the DAHP level in LC224 was much lower compared to QP478, which may suggest the *aroB* activity in LC224 driven by *tac* promoter was higher than that of QP478. Except for DAHP, LC224 accumulated a higher amount of metabolites in the shikimate pathway and fewer metabolites in the EDEMP cycle relative to QP478 (Fig. 6a, b), suggesting greater flux entering the shikimate pathway in LC224 and enabling greater muconate biosynthesis.

Although its precursor 3DHQ was not detected in any samples, quinic acid (quinate, QA) was substantially accumulated in LC224 (Fig. 6b), which may suggest an overflow of carbon resulting from overexpression of *aroB*. Shikimic acid (shikimate, SA) accumulation in LC224 is evidence of muconate biosynthesis through the *aroE* pathway, while SA accumulation was much lower in QP478 relative to LC224, which may be related to the *aroK* duplication in QP478. The accumulation of anthranilic acid in the culture media likely represents another case of overflow metabolism. More catechol (CAT) accumulated in LC224 (Fig. 6b), which could represent new bottlenecks associated with increased flux to muconate. Together, these results illustrate that engineering to generate LC224 broadly recapitulated the evolved strain QP478 and suggest additional targets for further improvement.

## Discussion

Technologies for the production of sustainable bio-based chemicals are needed to displace incumbent petrochemicals. Critical to this endeavor is the engineering of strains to convert lignocellulosic sugars such as glucose and xylose to product at industrially relevant titer, rate, and yield. In this work, the maximum theoretical molar yield of muconate from a mixture of glucose and xylose increased from ~40% with glucose alone to 50% when the xylose content in the mixture is between 33 and 40% (mol%), which is a relevant ratio in lignocellulosic hydrolysates (Fig. 1b, Supplementary Fig. 1). This was achieved by introducing the d-xylose isomerase pathway to supply E4P, and reintroducing *pgi-1* to enable the EDEMP cycle. ALE was used to identify additional targets to engineer a strain that ultimately achieved a 46% yield on a mixture of glucose and xylose (Fig. 5c), considerably higher than the 35.6% we had achieved previously with a strain engineered to convert glucose alone^32^.

During ALE, mutations in *xylE* arose in all the selected isolates (Figs. 2a and 3f) that improved growth on xylose. All five mutations are in the transmembrane domains of the transporter (Fig. 3f). Based on previous work in the same system^35^ and our own data showing that xylose metabolism was inhibited in the muconate-producing strain QP328 (Figs. 1c and 2b) but not in the non-muconate-producing analog LC345 (Supplementary Fig. 2a), we propose the mutations were a response to inhibitor(s) from the muconate-producing background strain. Further research to identify and characterize the potential inhibitors will be pursued in future work. Moreover, increased expression of PP_2569, a putative MFS transporter, enabled by a G→A point mutation in the promoter region, led to substantially higher muconate yield and lower biomass yield in LC100 (Fig. 2b, c), and metabolomics analysis suggested a metabolic flux redirect from the EDEMP cycle to the shikimate pathway (Fig. 3a). Intracellular and extracellular metabolomics analysis of strains LC091 and LC100 grown on xylose suggested PP_2569 may be able to export anthranilic acid, thereby reducing the intracellular accumulation of aromatic amino acids, which are known to inhibit native DAHP synthases. We also observed higher intensities of extracellular anthranilic acid in strains QP478 and LC224 compared to the unevolved strain QP328 (Fig. 6b). Mechanistic studies with PP_2569 may be of utility for further engineering.

ALE also resulted in a duplication of the genomic region from PP_5050–PP_5242. Within this region, we demonstrated that overexpression of *aroB* was necessary to reach high growth rates on xylose in LC224. In strain GB062, a strain previously engineered for improved conversion of glucose to muconate by deleting *hexR* in CJ522^3^, transcriptomics indicated that expression of *aroB* was already increased upon deletion of *hexR*^32^. In another study in which *P. putida* was engineered to produce PCA from glucose, overexpression of *aroB* did not contribute to improved production^45^. In strain LC100 cultivated on a mixture of glucose and xylose, however, AroB activity seemed to be rate limiting, since overexpression of *aroB* in LC224 improved growth and muconate production (Fig. 4d–i and Supplementary Fig. 10b, c). This may indicate that with xylose entering the non-oxidative pentose pathway, the supply of E4P was enhanced, leading to increased flux of carbon into the shikimate pathway via the condensation of E4P and PEP to DAHP. The improved level of DAHP made the next reaction, catalyzed by AroB, rate limiting, where it was not before. Overall, the engineering strategy shown here to improve flux of carbon into and through the shikimate pathway could be leveraged to improve the production of other shikimate-derived products from glucose and xylose in *P. putida*.

Our rationally engineered strain LC224 outperformed the evolved strain QP478 in growth on xylose (Fig. 4d). One potential reason could be the redundancy, complexity, and burden of the large duplication in QP478. Such duplications are likely enabled by recombination within similar sequences at two or more locations within the genome such that duplication of certain regions is favored or limited, ultimately limiting the ability of evolution to arrive at ideal outcomes within laboratory time scales. Genome engineering, however, can be used to make precise changes. Indeed, overexpression of *aroB* alone in LC224 outperformed QP478 (Fig. 4d–f and Supplementary Fig. 10a, c), which contains the entire PP_5050–PP_5242 duplication. This demonstrates the utility and power of ALE as a tool to identify targets for rational engineering.

Overexpression of *aroB* substantially improved the sugar utilization of LC224 relative to LC100 (Figs. 2d and 4h). In the metabolomics analysis, the intracellular DAHP level of LC224 is lower than the other two strains (Fig. 6). This may suggest that DAHP accumulation has a negative effect on sugar metabolism that can be relieved by *aroB* overexpression. Moreover, *aroB* overexpression alone in QP328, generating LC349, led to slightly lower muconate yield compared to LC224 on mixture of glucose and xylose (Supplementary Fig. 11d–f). Since restoring the G→A mutation in P_PP_2569_ in the QP478 strain did not have comparable effect to reverse engineering in LC091 in terms of varying the muconate yield (Fig. 2c and Supplementary Fig. 6g, h), these results together may suggest that *aroB* overexpression (resulting from duplication) and upregulation of PP_2569 have a similar effect on reducing feedback inhibition of DAHP synthases, which led to improved flux into muconate biosynthetic pathway.

Metabolomic analysis of our engineered strains provides early insights into future engineering efforts for further improving muconate production, beyond what we demonstrated here with LC224, which will be pursued in future studies. The quinate accumulation by this strain (Fig. 6b) may suggest an approach to improve its performance by overexpressing *aroQ* or deleting *quiA*. As for shikimate accumulation, overexpression of *aroB* and *aroK* did not improve the performance of LC168 relative to LC224, the equivalent strain overexpressing *aroB* alone (Fig. 4h and Supplementary Fig. 10c, e). This may suggest potential bottleneck(s) in the downstream steps of the pathway, especially considering the relatively high level of extracellular anthranilic acid (ANA) accumulated by QP478 (Fig. 6b). Insufficient conversion of chorismate (CSA) to 4-hydroxybenzoate (4HB) might be one cause of anthranilic acid accumulation. The gene *ubiC-C22* encoding chorismate pyruvate-lyase, which was previously engineered to reduce product inhibition, was driven in our strains by the relatively weak *lac* promoter, and a potential approach to accelerate muconate biosynthesis via shikimate in LC224 could be to overexpress *aroK* while simultaneously increasing the expression level of *ubiC-C22*.

Previously conducted techno-economic analysis for the conversion of glucose as well as glucose and pentose sugars^3^ indicated that the minimum selling price (MSP) of muconate would decrease substantially with increased yield and rate. Our engineered strain GB271 produced muconate from glucose at a 36% yield and a rate of 0.21 g L^−1^ h^−1^, which corresponds with an MSP around $3 kg^−1^ according to this model^32^. Here, LC224 achieved a nearly 50% yield at 0.28 g L^−1^ h^−1^ (Fig. 5b). This would reduce the MSP to around $2.2 kg^−1^, which is close to the $1.96 MSP previously predicted to be commercially viable^3^. The model suggests that the MSP can be further reduced by increasing the rate and yield. Considering the 50% molar yield is already at the theoretical maximum in our strain design, further rate increases will be key to MSP improvements.

In conclusion, this work demonstrates an effective strategy for producing muconate from glucose and xylose using *P. putida*. Considering the promising yield and titer of muconate from glucose and xylose, our strain LC224 could also represent a promising platform strain for the production of other shikimate pathway-derived compounds.

## Methods

### Plasmid construction

Q5^®^ High-Fidelity 2X Master Mix (New England Biolabs) was used for all polymerase chain reactions, followed by DpnI (New England Biolabs) digestion to remove the cell-derived plasmid template when necessary. NEBuilder HiFi Assembly Master Mix (New England Biolabs) was used for plasmid construction, followed by transformation into chemically competent NEB 5-α F’*I*^*q*^ *E. coli* (New England Biolabs), or into CopyCutter™ EPI400™ electrocompetent *E. coli* to increase plasmid yield, all according to manufacturer’s instructions. Transformants were selected on Lysogeny Broth (LB) agar (Lennox) plates supplemented with 50 μg mL^−1^ kanamycin (Sigma-Aldrich) and grown overnight at 37 °C. Correct constructs were confirmed by Sanger sequencing (GENEWIZ, Inc.). Some plasmids were synthesized (Twist Bioscience). Detailed plasmid construction information is described in Supplementary Data 2. Sequences for synthesized plasmids are in Supplementary Data 3. Oligos used in plasmid construction are listed in Supplementary Data 4.

### Strain construction

Gene deletions, insertions, and replacements were performed using the kanamycin-resistant gene *nptII* as a selection marker for the first-round homologous recombination of the plasmid into the chromosome and the sucrose-sensitive gene *sacB* as the counterselection marker for the second round of homologous recombination out of the chromosome^52^. Correct gene deletions, insertions, and replacements were identified by diagnostic colony PCR product based on differences in product size or presence using MyTaq^TM^ Red Mix (Bioline). For point mutation insertions and restorations, correct replacements were screened by comparing the intensity of the bands of the colony PCR products using primers of which the 3’ nucleotide annealed to the mutated nucleotide, followed by confirmation with Sanger sequencing.

Plasmids were transformed into *P. putida* via either electroporation or conjugation. For electroporation, *P. putida* strains were inoculated from a glycerol stock into LB medium, and cultivated overnight at 30 °C, 225 rpm. Electrocompetent cells were prepared by centrifuging 2.5 mL overnight culture and washing in 300 mM sucrose three times, followed by resuspending cells in 50 μL 300 mM sucrose^53^. Plasmids for electroporation were constructed using the backbone plasmid pK18sB^54^, and electroporated in 0.1 cm cuvette using a Gene Pulser Xcell (Bio-Rad) with the setting 1.6 kV, 25 μF, 200 ohms. The whole process was performed at room temperature. For conjugation, plasmids were built based on backbone plasmid pK18msB which contains the R4P *oriT*, and were transferred from donor *E. coli* S17-1 cells^55^ to the recipient *P. putida* strains. The plasmid-containing donor *E. coli* S17-1 strain was inoculated into LB medium supplemented with 50 μg mL^−1^ kanamycin and cultivated in a shaking incubator at 37 °C, 225 rpm overnight; the recipient *P. putida* strain was inoculated into LB medium and cultivated in a shaking incubator overnight at 30 °C, 225 rpm. Subsequently, 1 mL overnight donor and recipient cells were centrifuged at 5000×*g* for 2 min and washed with LB medium twice, then the pellets were resuspended and 400 µL of each were mixed in one microcentrifuge tube and centrifuged again. The mixed pellets were resuspended using 50 µL LB medium and dropped onto a LB plate with two antibiotics at low concentration: 10 μg mL^−1^ chloramphenicol to inhibit cell growth of *E. coli* S17-1, and 5 μg mL^−1^ kanamycin to inhibit cell growth of *P. putida*. The plate was incubated at 30 °C for 6–10 h to allow for conjugation, after which the cells were streaked for single colonies on the same plate and incubated at 30 °C overnight. Single colonies on the LB plate containing low concentration antibiotics were restreaked to a new LB plate with 100 μg mL^−1^ chloramphenicol, which *P. putida* is naturally resistant to, and 50 μg mL^−1^ kanamycin, to select for transconjugants. All strains used in this study are described in Table 1, detailed strain construction information is described in Supplementary Data 5.

### Shake flask and plate reader experiments

For shake flask and growth curve experiments, seed cultures were inoculated from glycerol stocks into 14-mL round bottom Falcon^®^ tubes containing 5 mL of LB Miller medium and incubated overnight at 30 °C and 225 rpm. Overnight cultures were then inoculated into a 125-mL baffled shake flask containing 10 mL LB medium to an initial OD_600_ of 0.2. These second seed cultures were cultivated at 30 °C at 225 rpm for 4 h to reach an OD_600_ of ~2. The second seed cultures were washed twice with M9 salts (6.78 g L^−1^ Na_2_HPO_4_, 3 g L^−1^ KH_2_PO_4_, 0.5 g L^−1^ NaCl, 1 g L^−1^ NH_4_Cl) and then inoculated into a 125-mL baffled shake flask containing 25 mL modified M9 minimal medium (6.78 g L^−1^ Na_2_HPO_4_, 3 g L^−1^ KH_2_PO_4_, 0.5 g L^−1^ NaCl, 1 g L^−1^ NH_4_Cl, 2 mM MgSO_4_, 100 µM CaCl_2_, 18 µM FeSO_4_) supplemented with either 30 mM xylose, 30 mM glucose, or mixture of 30 mM glucose and 15 mM xylose, to an initial OD_600_ of 0.1. The molar ratio of glucose and xylose in mixed substrates is 2:1, which is the ratio typical of corn stover hydrolysates^56^. All growth curves were characterized on Bioscreen C Pro analyzers (Growth Curves US) using 300-µL cultures inoculated as described for the shake flasks above. Shake flasks and plate reader data were plotted and analyzed using GraphPad Prism version 8.4.2. Absolute growth rate (*μ*_A_) and growth lag (*λ*) were calculated based on Gompertz equation using default settings of the FittR tool deposited in GitHub (https://github.com/scott-saunders/growth_curve_fitting)^57^. In some cases, death phase was removed from the growth curve to fit the model.

The pH values of flasks were monitored at each sampling time point using a mini pH meter (HORIBA LAQUAtwin pH-33), and when necessary, 1 N NaOH was used to adjust the pH to 7. For shake-flask experiments, yields were calculated at the time point where maximum muconate concentration was detected.

### Metabolic modeling

A core-carbon metabolic model of *P. putida*^3^, which considers the reaction shown in Fig. 1a, was extended with reactions from the *aroE* synthetic route, using stoichiometry adapted from a genome-scale model of *P. putida* KT2440^58^. Yield calculations were performed assuming no ATP maintenance requirement by optimizing the muconate flux while varying the xylose fraction in the medium. Calculations were performed using the cobrapy library (version 0.22.1) in Python 3.8.10^59^.

### Adaptive laboratory evolution with xylose as the sole carbon source

To conduct ALE, strain QP328 was first inoculated in LB Miller medium from a glycerol stock and grown overnight as a seed culture. Cells from the overnight culture were then washed with M9 salts twice and resuspended in M9 salts. The washed cells were inoculated to an initial OD_600_ 0.1 in a culture tube containing 5 mL M9 medium with 10 mM xylose as a sole carbon source and cultivated at 30 °C with shaking at 225 rpm. Serial passaging was performed by transferring 1% (v/v) of the culture into fresh medium when growth was observed, which initially took up to around two weeks of cultivation. The number of generations was calculated based on the OD_600_ value, according to Eq. (1):^32^

1 $${{{{{\rm{Number}}}}}}\,{{{{{\rm{of}}}}}}\,{{{{{\rm{generations}}}}}}={{{{{\rm{ln}}}}}}({{{{{\rm{OD}}}}}}_{{{{{\rm{final}}}}}}/{{{{{\rm{OD}}}}}}_{{{{{\rm{initial}}}}}})/{{{{{\rm{ln}}}}}}(2)$$

Initial, periodic intermediate, and final populations were preserved as glycerol stocks.

### Muconate, glucose, and xylose analyses

*cis,cis*- and *cis,trans*-muconic acid isomers were analyzed by preparing a unique standard of *cis,trans*-muconic acid to accomplish accurate quantitation of both muconic acid isomers^60^. Separation and detection were achieved using the following chromatographic conditions. Samples and standards were analyzed using an Agilent 1290 series UHPLC (Agilent Technologies) coupled with a diode array detector (DAD) and a Phenomenex Luna C18(2)-HST column 2.5 μm, 2 ×100 mm column. An injection volume of 1 μL was injected onto the column in which the temperature was held constant at 45 °C. Muconic acid isomers were monitored and quantified at the wavelength 265 nm with a quantitation range of 1 ppm to 500 ppm. A gradient of 0.16% formic acid in water (A) and acetonitrile (B) was utilized at a flow rate of 0.5 mL min^−1^. Chromatographic separation of analytes was attained using the following gradient program: initial (*t*_0_) to *t* = 1 min: A-100% and B-0%; ramp to A-50% and B-50% from *t* = 1–7.67 min; ramp to A-30% and B-70% from *t* = 7.67–9.33 min and held until 10.67 min. At 10.68 min, the gradient was returned to A-100% and B-0% and maintained isocratic for a total run time of 13 min. A calibration verification standard was run every 10–15 samples to ensure detector stability. Glucose was quantified by HPLC with refractive index detection coupled with an Aminex HPX-87H column (Bio-Rad)^61^, while xylose was similarly and simultaneously quantified. Pure standards were purchased from Sigma-Aldrich.

### Quantitative reverse-transcription PCR

For quantitative reverse-transcription PCR (RT-qPCR) of PP_2569 in strains LC091 and LC100, cells were cultivated in shake flasks with M9 and 30 mM xylose according to the shake flasks protocol mentioned above. For RT-qPCR of PP_4302 in strains LC100 and LC224, cells were cultivated in shake flasks with LB medium. Cells were harvested in mid-log phase, and were broken using ﻿TRI^®^ Reagent (Sigma, T9424), followed by RNA miniprep using Direct-zol^TM^ RNA miniprep kit (Zymo Research, R2052) following the protocol. The extracted total RNAs were then digested using DNase I (Zymo Research, E1009-A) and purified using RNA Clean & Concentrator^TM^−5 kit (Zymo Research, R1014). RNA concentrations were determined using NanoDrop^TM^ one (Thermo Scientific) at 260 nm, 500 ng total RNA of each sample were added for reverse transcription, using the iScript™ Reverse Transcription Supermix (Bio-Rad), to synthesize cDNA. RT-qPCR was conducted using KiCqStart^®^ SYBR^®^ Green qPCR ReadyMix (Sigma-Aldrich), ﻿with the 7500 Fast Real-Time PCR System (Applied Biosystems).

Housekeeping gene *rpoD* was employed as a reference control to normalize different samples^62^. Primers oLC-0109 and oLC-0110 were used for amplifying *rpoD*; oLC-0107 and oLC-0108 were used for amplifying PP_2569; oLC-0353 and oLC-0354 were used for amplifying PP_4302. We used the 2^-ΔΔCt^ method to quantify transcriptional levels between samples^63^.

### Bioreactor cultivations

To evaluate strains QP478 and LC224 in bioreactors, the strains were inoculated from glycerol stocks in 250 mL baffled flasks containing 50 mL of LB (Miller) and incubated at 30 °C and 225 rpm for 16 h. Seed cultivations were conducted in duplicate for each strain and each replicate was utilized to inoculate independent bioreactors. The cells were centrifuged (5000×*g*, 10 min), the supernatant was discarded, and the cells resuspended in 5 mL of modified M9. The modified M9 contained 13.56 g L^−1^ Na_2_HPO_4_, 6 g L^−1^ KH_2_PO_4_, 1 g L^−1^ NaCl, 2 g L^−1^ (NH_4_)_2_SO_4,_ 2 mM MgSO_4_, 100 μM CaCl_2_, and 36 μM FeSO_4._

Cells were inoculated in 0.5 L bioreactors (BioStat-Q Plus, Sartorius Stedim Biotech) at an initial OD_600_ of 0.2. The batch phase consisted of growth on 300 mL of modified M9 with 10.6 g L^−1^ glucose and 4.4 g L^−1^ xylose, which mimics the sugar ratio in sugar hydrolysates from corn stover^56^. The fed-batch phase was initiated when the sugar concentration was approximately 7 g L^−1^, at which point sugars were fed to maintain sugar concentrations between ~2–10 g L^−1^ by manually modifying feeding rates. The feeding solution contained 353 g L^−1^ glucose and 147 g L^−1^ of xylose, and its pH was adjusted to pH 7 with NaOH. The bioreactors were controlled at pH 7 by the addition of 4 N NH_4_OH, at 30 °C, and air was sparged at 1 vvm. The initial agitation speed in the batch phase was 350 rpm. When the dissolved oxygen (DO) reached a value of 30%, it was automatically controlled at that level by automatic agitation adjustments. Samples were taken periodically to evaluate bacterial growth and to analyze sugar concentrations and muconate.

### Metabolomic analysis

Cell pellets and supernatant samples were collected separately by centrifugation of 1 mL of shake-flask cultures grown on mixture of glucose and xylose at the ﻿mid-log phase (OD_600_ ~1.0), and the samples were frozen at −80 °C until analysis. Cell pellets were lyophilized, and the intracellular metabolites were extracted from 3 mg of dried biomass using a solvent mixture of chloroform/methanol/water^64^, and both aqueous and organic layers were transferred to a new vial and dried completely. The extracellular metabolites were prepared by drying the supernatant samples under vacuum before the analysis. The metabolites were chemically derivatized based on the method below^65^. In order to protect carbonyl groups and decrease the formation of tautomeric isomers, a solution of methoxyamine (20 µL of a 30 mg mL^−1^ stock in pyridine) was added to each dried extract. Samples were then incubated at 37 °C with shaking for 90 min. To remove acidic protons in hydroxyl-, amino- and carboxyl- groups, a trimethylsylilation reaction was used, specifically *N*-methyl-*N*-(trimethylsilyl) trifluoroacetamide with 1% trimethylchlorosilane (80 µL) was added to each vial. Samples were again incubated at 37 °C with continuous shaking for 30 min. After incubation was completed, the vials were centrifuged, and the liquid transferred to a small volume glass insert. The samples were analyzed by gas chromatography-mass spectrometer (GC-MS) using a HP-5MS column (30 m × 0.25 mm × 0.25 µm; Agilent Technologies) for untargeted analyses. A volume of 1 µL was injected of each sample in splitless mode, and the helium gas flow rate was established by using the Retention Time Locking function based on the elution time of deuterated myristic acid (Agilent Technologies, Santa Clara, CA). The injection port temperature was maintained at 250 °C throughout the analysis. The GC oven gradient started with a temperature of 60 °C held for 1 min after injection, followed by an increase to 325 °C at a rate of 10 °C min^−1^, ending with a 10 min hold at 325 °C. The collection of MS data covered the mass range 50–600 *m/*z. A mixture of fatty acid methyl esters (FAMEs, C8-C28) was analyzed alongside every batch of samples to be able to perform retention index alignment in the subsequent data analysis. GC-MS data files were converted to CDF format, and they were deconvoluted and aligned using the software Metabolite Detector 2.5^66^. Identification of metabolites was done by matching with PNNL metabolomics databases—augmented version of Fiehn database^67^. This database contains mass spectra and retention index information of over 1000 authentic chemical standards and has been cross-checked with commercial GC-MS databases such as NIST20 spectral library and Wiley 11th version GC-MS databases^68,69^. Three unique fragment ions were selected and used to integrate peak area values, and a few metabolites were curated manually when necessary.

### Reporting summary

Further information on research design is available in the Nature Research Reporting Summary linked to this article.

## Supplementary information

Supplementary Information

Description of Additional Supplementary Files

Supplementary Data 1

Supplementary Data 2

Supplementary Data 3

Supplementary Data 4

Supplementary Data 5

Reporting Summary

## Data Availability

Whole-genome sequencing data that support the findings of this study have been deposited in the NCBI SRA database with the accession number PRJNA783062. PP_2569 can be accessed in Uniprot database using the link https://www.uniprot.org/uniprotkb/Q88JS8/entry. Source data are provided with this paper.

## Source data

Source Data
