# Supplementary material for: Muconic acid production from glucose and xylose in Pseudomonas putida via evolution and metabolic engineering
Source: Nat Commun. 2022 Aug 22;13:4925. doi: 10.1038/s41467-022-32296-y (PMC9395534; doi:10.1038/s41467-022-32296-y)
Supplement: Supplementary file 1 — Supplementary Information [file 41467_2022_32296_MOESM1_ESM.pdf]

Muconic acid production from glucose and xylose in *Pseudomonas putida* via evolution and metabolic engineering

Ling *et al*.

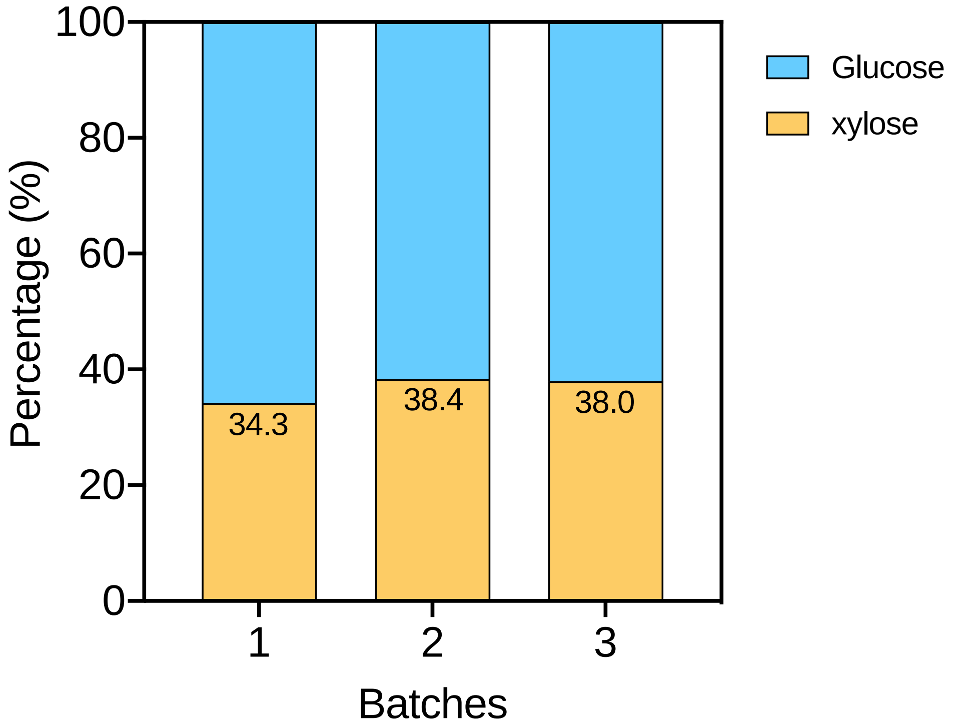

Supplementary Figure 1. **Xylose content relative to glucose by mole percent in corn stover hydrolysate.** Three batches are shown. Batch 1 was reported by Elmore et al.,[1] and batch 2 and 3 were from our recent samples.

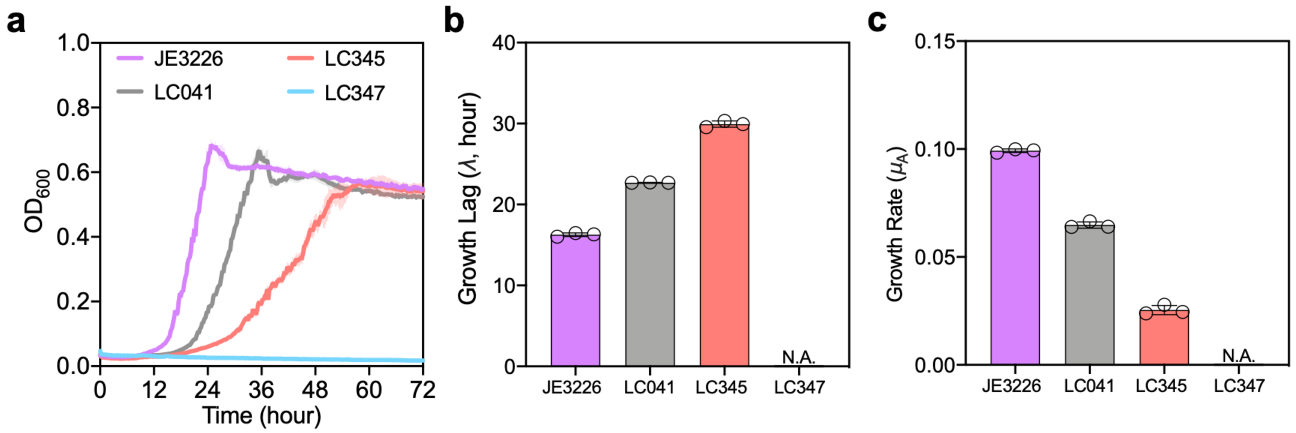

Supplementary Figure 2. **Investigation of the function of *pgi-1* and *pgi-2* on xylose metabolism. a.** Growth curves of JE3226, LC041 (JE3226 *Δpgi-1*), LC345 (JE3226 *Δpgi-2*), and LC347 (JE3226 *Δpgi-1 Δpgi-2*) on M9 medium supplemented with 10 mM xylose in a plate reader. **b, c.** Growth lag (λ) and absolute growth rate (μ_A) from panel a were extracted and plotted separately for better comparison, error bars represent standard derivation in these values of three independent growth curves. Source data are provided as a Source Data file.

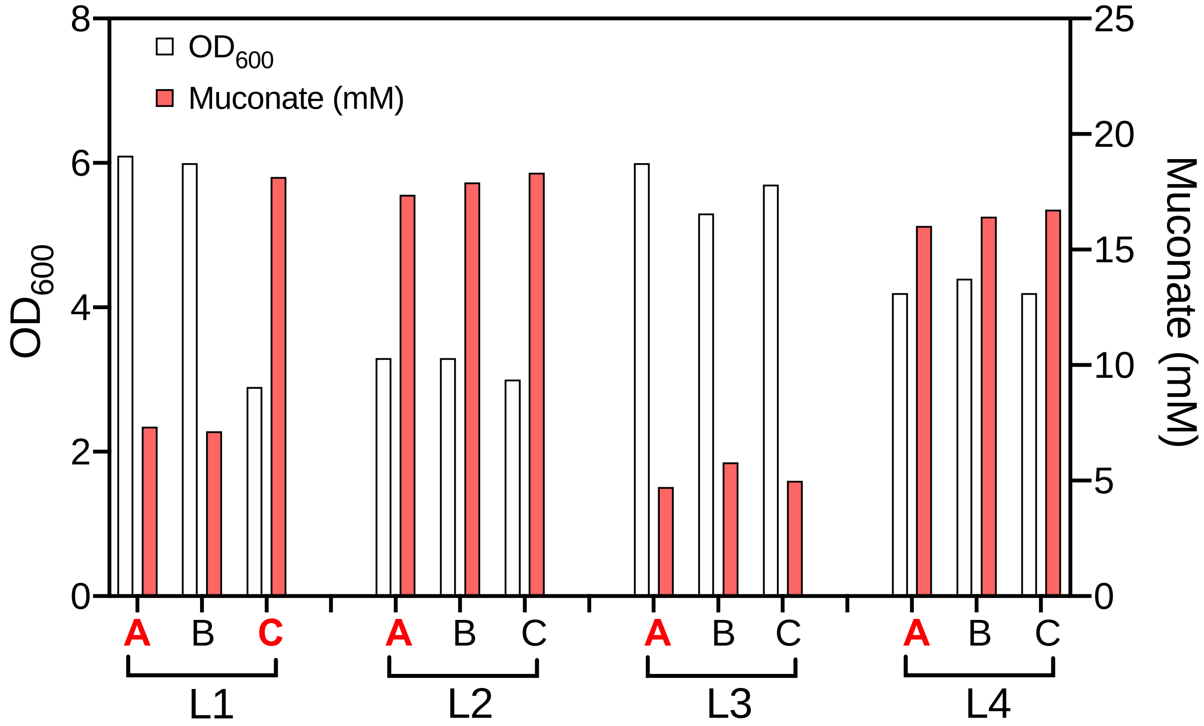

Supplementary Figure 3. **Evaluation of the evolved isolates in shake flasks.** 12 isolates from 4 independent lineages (L1-L4) were evaluated in shake flasks containing M9 medium supplemented with 30 mM glucose and 25 mM xylose. Cells were cultivated for 72 hours without adjusting pH, and final OD_{600} values and muconate concentration are shown. Isolates labeled in red were named isolate 1-5 from left to right. Source data are provided as a Source Data file.

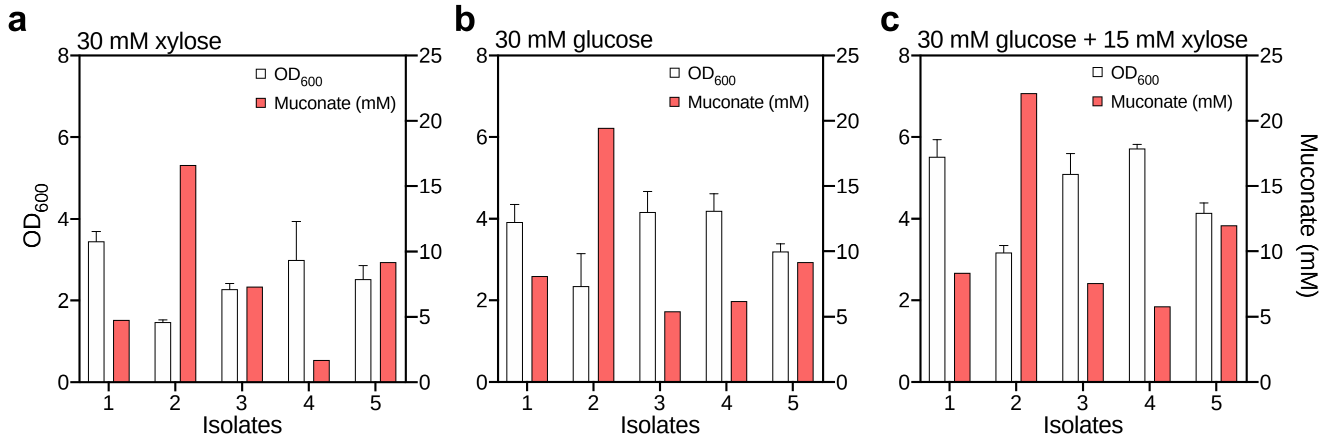

Supplementary Figure 4. **Shake flask results of the 5 evolved isolates on different substrates.** Final OD_{600} and muconate concentrations of shake flask cultures grown on M9 medium supplemented with **a.** 30 mM xylose, **b.** 30 mM glucose, **c.** 30 mM glucose and 15 mM xylose. Experiments were performed in quadruplicate and samples were collected and OD_{600} were measured at the 72-hour final time point. The error bars in OD_{600} values represent standard deviations. Only one out of the quadruplicates were analyzed for muconate quantification. Source data are provided as a Source Data file.

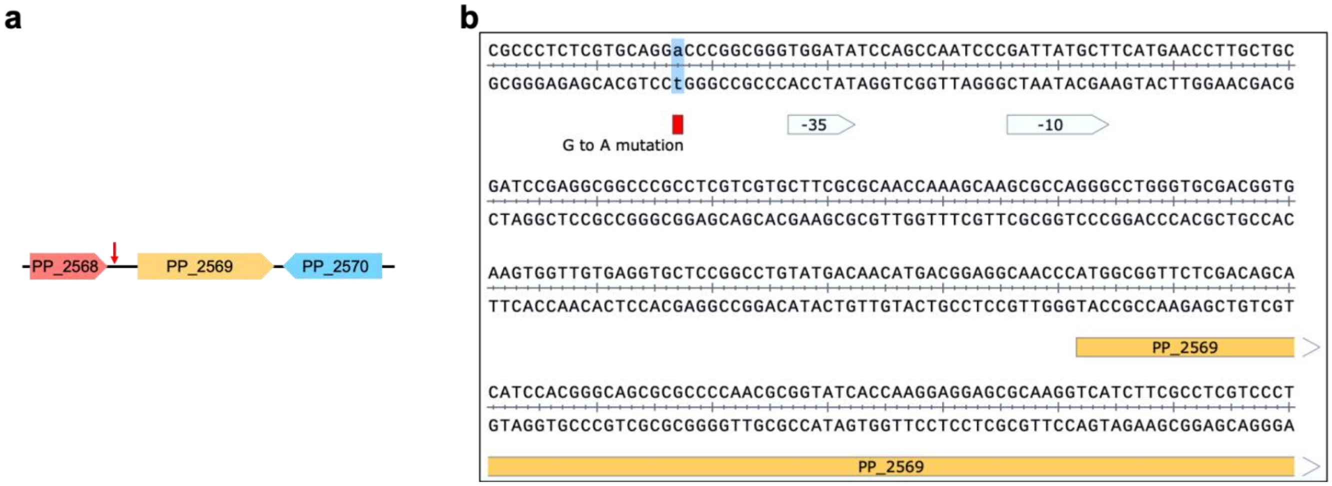

Supplementary Figure 5. **Genetic context of G to A point mutation in the promoter region of PP_2569. a.** Location of the G to A mutation in the intergenic region, red arrow represents the mutation. **b.** Sequence-level detail of the mutation, the -10 and -35 elements were predicted using BPROM σ70 promoter prediction software.[2]

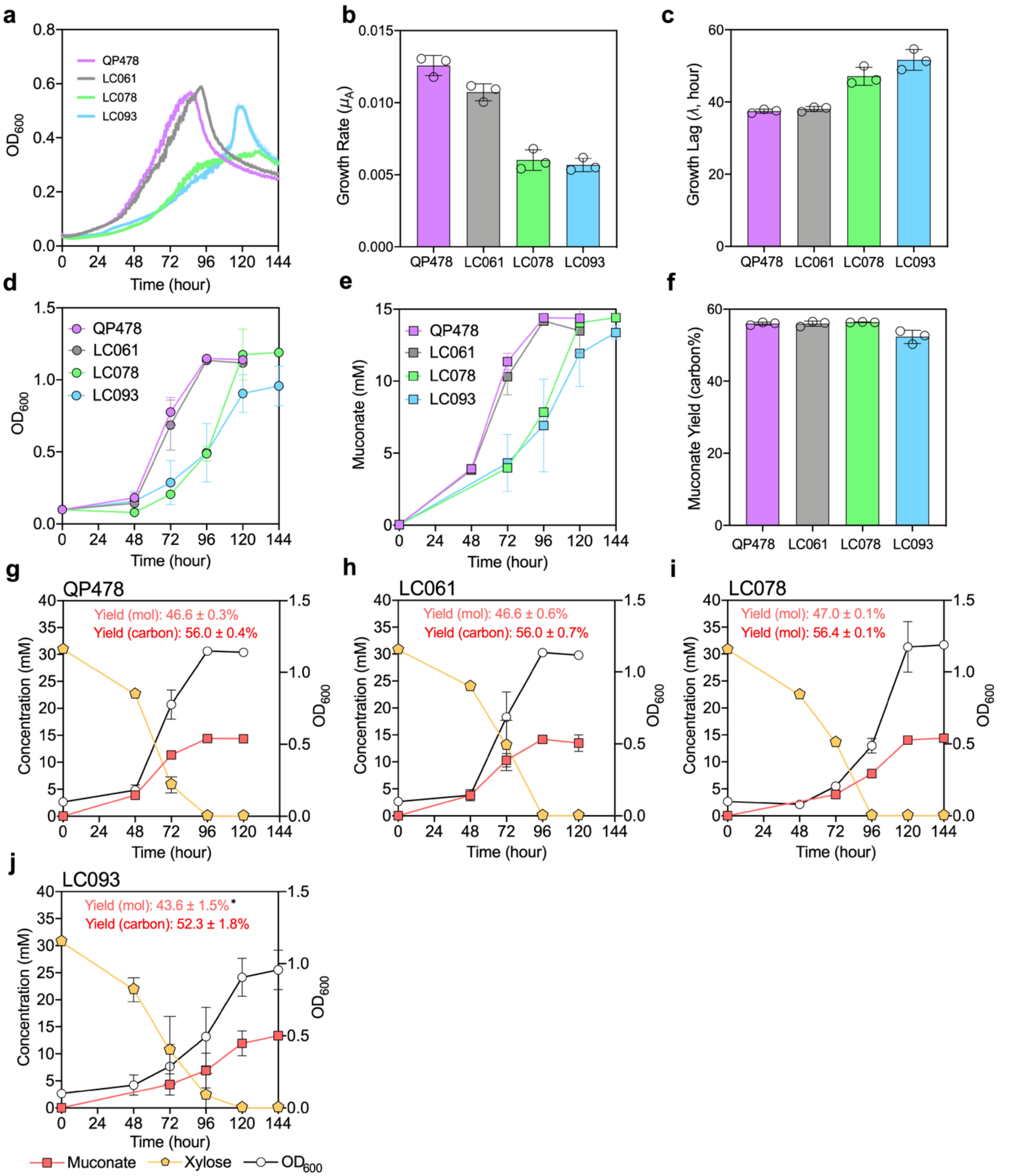

Supplementary Figure 6. Characterization of the point mutations by restoring the wild-type sequences in the evolved strain QP478. a. Growth curves of QP478, LC078 (QP478 with *xylE-A455V* restored to wild-type A), LC093 (QP478 with *xylE-A62V* restored to wild-type A), and LC061 (QP478 with G→A in P_{PP_2569} restored to wild-type G) on M9 medium supplemented with 30 mM xylose in a plate reader. **b-c.** Parameters extracted from panel **a**, including growth lag (λ) and absolute growth rate (μ_A), both are the average values of at least 3 independent growth curves. **d-f.** Comprison of the cell growth (**d**), muconate production (**e**) and final muconate carbon yield (**f**) of strains QP478, LC061, LC078 and LC093 in shake flask experiments on M9 medium containing 30 mM xylose. **g-j.** Profiles of shake flask experiments mentioned above in panels **d-f**. For shake flask experiments, % molar yield was calculated as (mM muconate/mM xylose × 100), % carbon yield was calculated as (mM muconate × 6/mM xylose × 5 × 100). Yields of LC078, LC093, and LC061 were compared to QP478 using two-tailed student *t*-test, only LC093 showed significance ($p < 0.05$). Error bars here represent the standard deviations of three biological replicates. Source data are provided as a Source Data file.

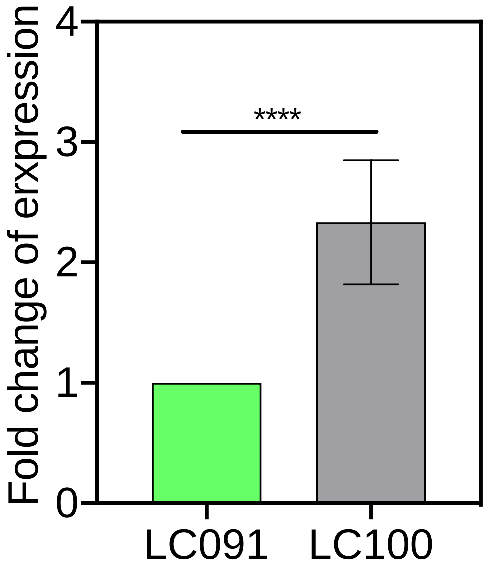

Supplementary Figure 7. **RT-qPCR of PP_2569 in strains LC091 and LC100 grown on xylose.** RT-qPCR analysis of PP_2569 expression levels in strains LC091(QP328 *xylE-A62V, A455V*) and LC100 (QP328 *xylE-A62V, A455V* P_{PP_2569} G→A) grown on M9 medium supplemented with 30 mM xylose. Both strains were sampled in mid log phase. Data represent the mean of 2 biologically replicates and 3 technical replicates. Error bars here represent the standard deviation. Two-tailed Student's *t*-tests were performed to determine the statistical significance, and $p <$ 0.0001. Source data are provided as a Source Data file.

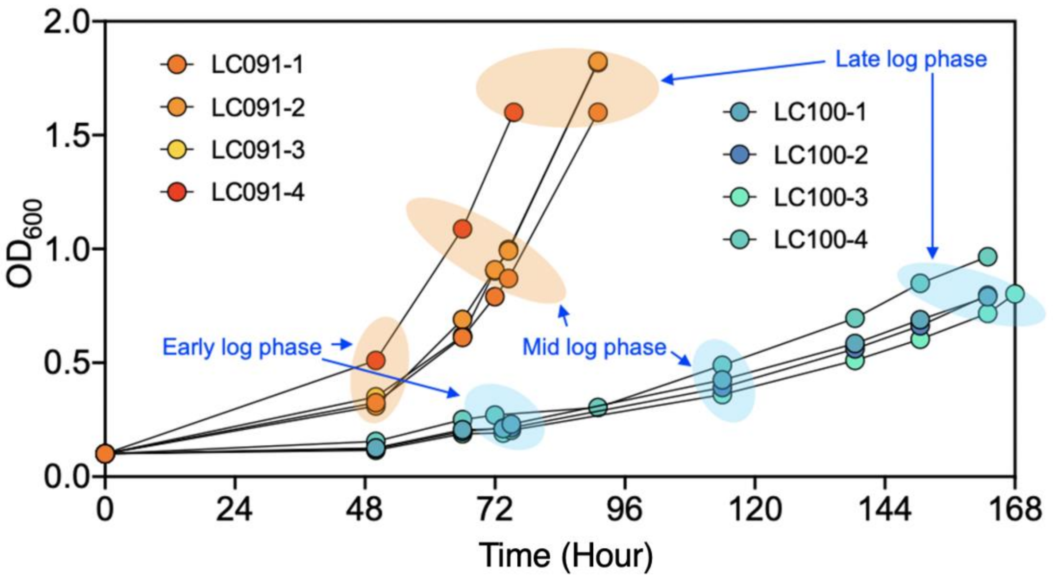

Supplementary Figure 8. **Sampling timepoints for metabolomics analysis of strains LC091 and LC100.** Strains were grown on M9 medium supplemented with 30 mM xylose. Shake flasks were conducted in quadruplicates. The definition of early log phase is around ¼ of the maximum OD_{600}, mid log phase is around ½ of the maximum OD_{600}, and late log phase is around ¾ of the maximum OD_{600}. Source data are provided as a Source Data file.

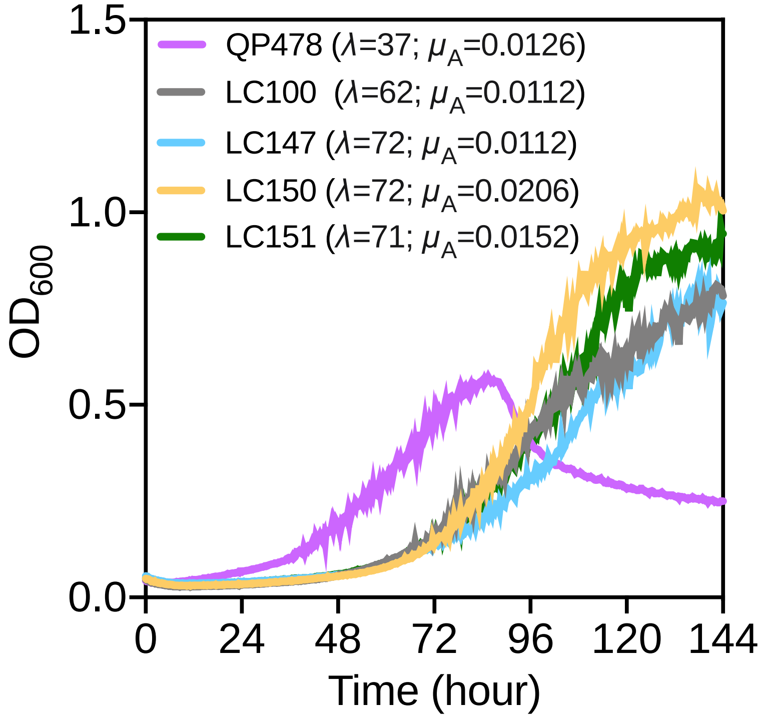

Supplementary Figure 9. Growth curves of strains QP478, LC100 (QP328 *xylE-A62V, A455V* P_{PP_2569} G→A), LC147 (LC100 Δ*pykF*::P_{lac}:*gpmI*), LC150 (LC100 Δ*pykF*::P_{tac}:*maeB*), and LC151 (LC100 Δ*pykF*::P_{tac}:*rpiA*) on M9 medium supplemented with 30 mM xylose in a plate reader, to evaluate the effect of candidate genes overexpression in strain LC100. Error bars here represent the standard deviation of three biological replicates. Source data are provided as a Source Data file.

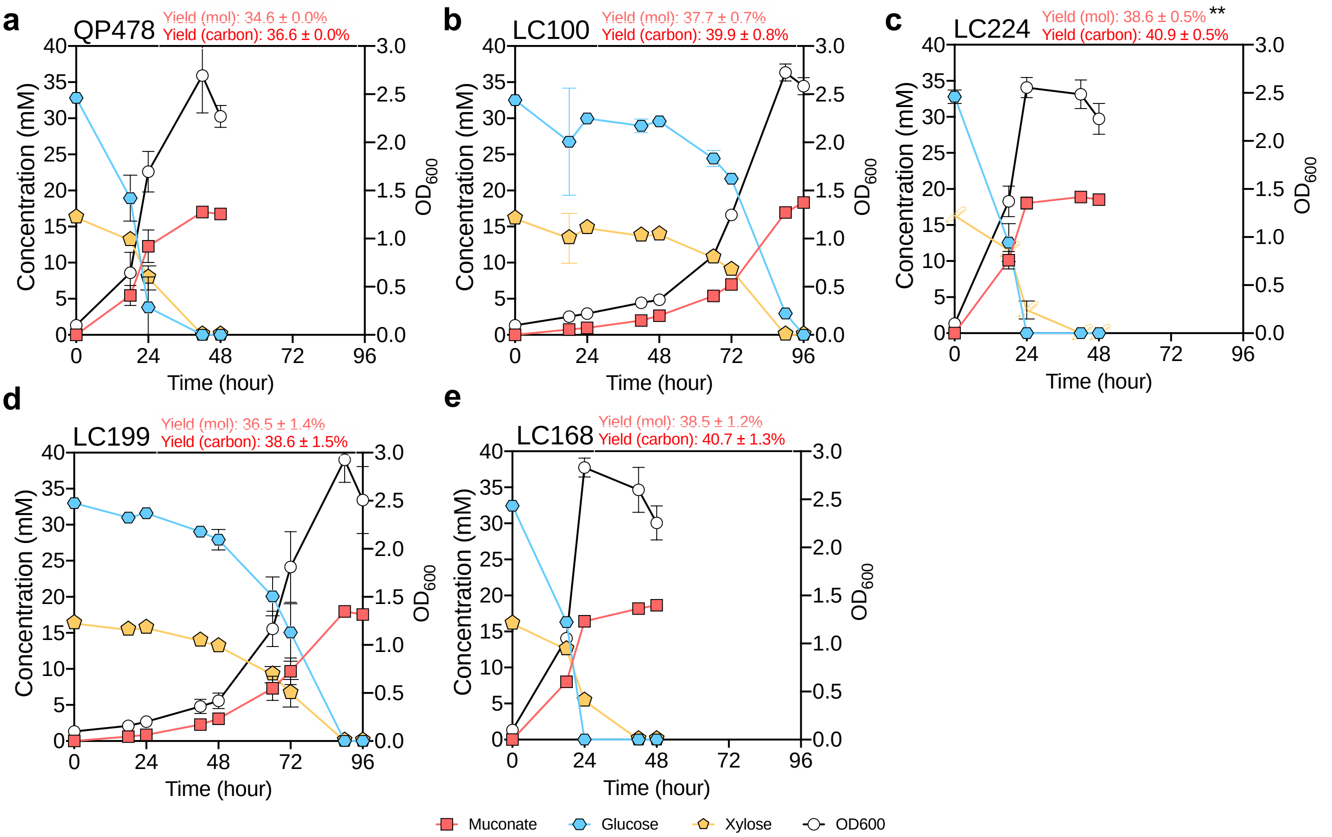

Supplementary Figure 10. **Shake flasks profiles from strains QP478, LC100, LC199, LC224 and LC168.** Profiles of shake flask experiments examining conversion of 30 mM glucose and 15 mM xylose to muconate in **a.** Strains QP478 **b.** LC100 **c.** LC224 (LC100 Δ*pykF*::P_{tac}:*aroB*) **d.** LC199 (LC100 Δ*pykF*::P_{tac}:*aroK*) and **e.** LC168 (LC100 Δ*pykF*::P_{tac}:*aroK*:*aroB*). pH values were monitored and, if necessary, adjusted to 7 at each sampling time point. % Molar yield was calculated as [mM muconate/mM (glucose + xylose) × 100], % carbon yield was calculated as [mM muconate × 6/mM (glucose × 6 + xylose × 5) × 100]. Molar yield of LC224 was compared to QP478 using two-tailed student *t*-test, with ** representing a statistically significant difference between the strains ($p < 0.01$). Error bars here represent the standard deviation of three biological replicates. Source data are provided as a Source Data file.

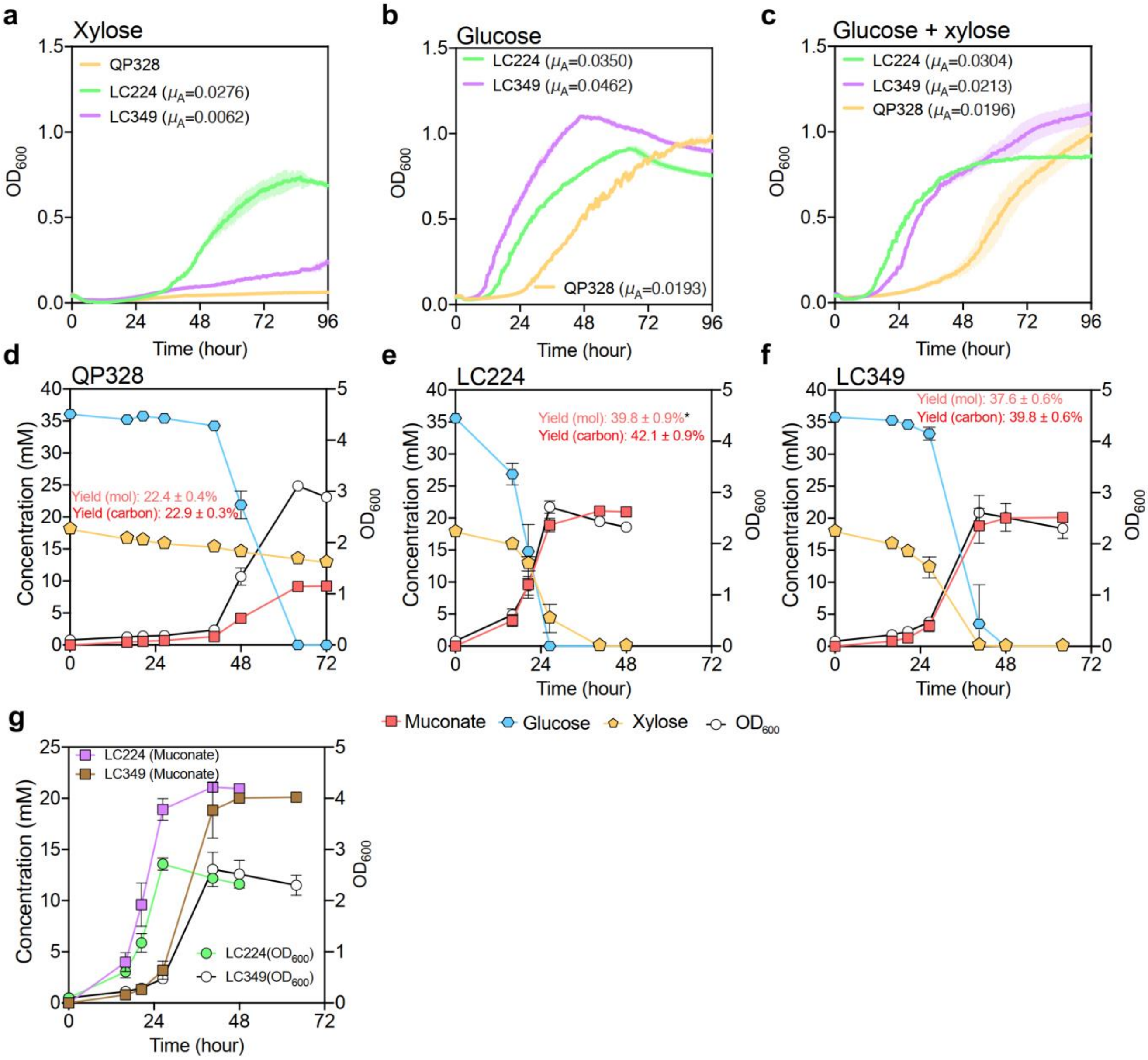

Supplementary Figure 11. **Evaluation of LC349 comparing to QP328 and LC224. a-c.** Growth curves of strains LC349, QP328 and LC224, on M9 medium supplemented with various substrates, including 30mM xylose (**a**), 30mM glucose (**b**), and 30 mM glucose + 15mM xylose (**c**). μ_A represents absolute growth rate, the average values of at least 3 independent growth curves. **d-f.** Shake flasks experiments comparing strains LC349, QP328 and LC224 on M9 medium supplemented with 30 mM glucose and 15 mM xylose. % Molar yield was calculated as [mM muconate/mM (glucose + xylose) × 100], % carbon yield was calculated as [mM muconate × 6/mM (glucose × 6 + xylose × 5) × 100]. Molar yield of LC224 was compared to LC349 using two-tailed student *t*-test, with * representing a statistically significant difference between the strains ($p < 0.05$). **g.** Muconate production and OD_{600} values of LC224 and LC349 in panels **e** & **f** were plotted separately, for better comparison of the muconate production during the cultivation time. Error bars here represent the standard deviation of three biological replicates. Source data are provided as a Source Data file.

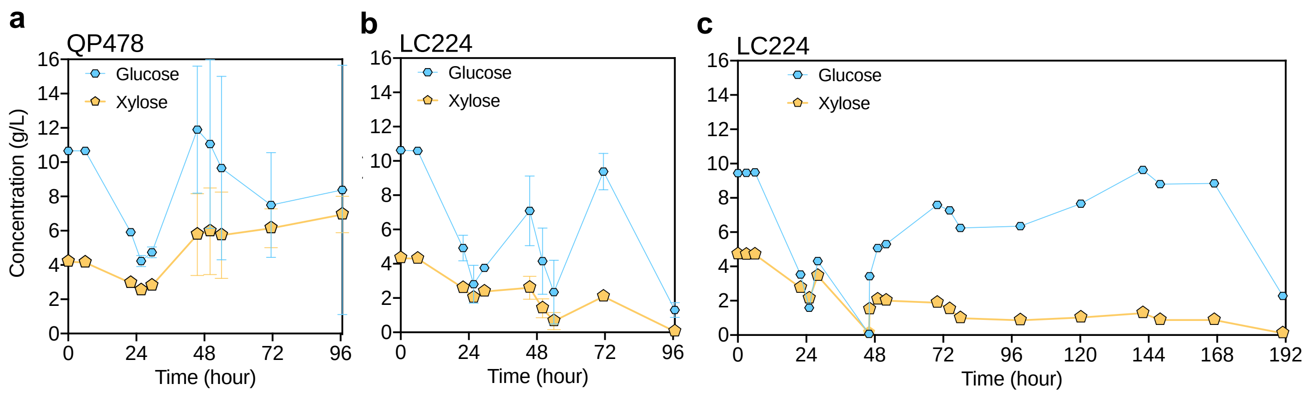

Supplementary Figure 12. **Glucose and xylose concentrations during the fed-batch bioreactor cultivations**. Sugar profiles are shown for **a,** QP478 and **b,** LC224 cultivated for 96.6 hours, and **c** LC224 cultivated for 191 hours. Results of **a** and **b** show the average of biological duplicates, results of **c** are from a single cultivation. Error bars represent the absolute difference between replicates. Source data are provided as a Source Data file.

Supplementary references

1. Elmore JR, *et al*. Engineered *Pseudomonas putida* simultaneously catabolizes five major components of corn stover lignocellulose: Glucose, xylose, arabinose, *p*-coumaric acid, and acetic acid. *Metab. Eng.* **62**, 62-71 (2020).
2. Salamov VSA, Solovyevand A. Automatic annotation of microbial genomes and metagenomic sequences. *Metagenomics and its applications in agriculture, biomedicine and environmental studies Hauppauge: Nova Science Publishers*, 61-78 (2011).
