# Supplementary material for: Muconic acid production from glucose and xylose in Pseudomonas putida via evolution and metabolic engineering
Source: Nat Commun. 2022 Aug 22;13:4925. doi: 10.1038/s41467-022-32296-y (PMC9395534; doi:10.1038/s41467-022-32296-y)
Supplement: Supplementary file 2 — Description of Additional Supplementary Files [file 41467_2022_32296_MOESM2_ESM.pdf]

Description of Additional Supplementary Files

File Name: Supplementary Data 1
Description: The mutations identified in QP478.

File Name: Supplementary Data 2
Description: Construction details for plasmids used in this study.

File Name: Supplementary Data 3
Description: Plasmid sequences in this study.

File Name: Supplementary Data 4
Description: Oligonucleotides used in this study.

File Name: Supplementary Data 5
Description: Strain construction details in this study.
