# Supplementary material for: Muconic acid production from glucose and xylose in Pseudomonas putida via evolution and metabolic engineering
Source: Nat Commun. 2022 Aug 22;13:4925. doi: 10.1038/s41467-022-32296-y (PMC9395534; doi:10.1038/s41467-022-32296-y)
Supplement: Supplementary file 8 — Reporting Summary [file 41467_2022_32296_MOESM8_ESM.pdf]

nature portfolio

Corresponding author(s): Adam M. Guss, Christopher W. Johnson, Gregg T. Beckham

Last updated by author(s): Jul 5, 2022

Reporting Summary

Nature Portfolio wishes to improve the reproducibility of the work that we publish. This form provides structure for consistency and transparency in reporting. For further information on Nature Portfolio policies, see our Editorial Policies and the Editorial Policy Checklist.

Statistics

For all statistical analyses, confirm that the following items are present in the figure legend, table legend, main text, or Methods section.

n/a	Confirmed	
☐	☒	The exact sample size (*n*) for each experimental group/condition, given as a discrete number and unit of measurement
☐	☒	A statement on whether measurements were taken from distinct samples or whether the same sample was measured repeatedly
☐	☒	The statistical test(s) used AND whether they are one- or two-sided<br>*Only common tests should be described solely by name; describe more complex techniques in the Methods section.*
☒	☐	A description of all covariates tested
☐	☒	A description of any assumptions or corrections, such as tests of normality and adjustment for multiple comparisons
☐	☒	A full description of the statistical parameters including central tendency (e.g. means) or other basic estimates (e.g. regression coefficient) AND variation (e.g. standard deviation) or associated estimates of uncertainty (e.g. confidence intervals)
☐	☒	For null hypothesis testing, the test statistic (e.g. *F*, *t*, *r*) with confidence intervals, effect sizes, degrees of freedom and *P* value noted<br>*Give P values as exact values whenever suitable.*
☒	☐	For Bayesian analysis, information on the choice of priors and Markov chain Monte Carlo settings
☒	☐	For hierarchical and complex designs, identification of the appropriate level for tests and full reporting of outcomes
☒	☐	Estimates of effect sizes (e.g. Cohen's *d*, Pearson's *r*), indicating how they were calculated

Our web collection on statistics for biologists contains articles on many of the points above.

Software and code

Policy information about availability of computer code

Data collection	A previously developed core-carbon metabolic model (Johnson CW, et al., Joule, 2019) was extended with reactions from the aroE synthetic route, using stoichiometry adapted from a genome-scale model of P. putida KT2440 (Nogales J, et al., bioRxiv, 2017).
Data analysis	Shake flasks and plate reader data were plotted and analyzed using GraphPad Prism version 8.4.2; next generation sequencing data were analyzed using Geneious Prime 2020.0.4; yield calculations in Figure 1b were performed using the cobrapy library (version 0.22.1) in Python 3.8.10. Growth rate and lag time were calculated using growth curve fitting tool FittR v1.0, which is deposited in GitHub: https://github.com/scott-saunders/growth_curve_fitting. Promoter was predicted by the BPROM σ70 promoter prediction program: http://www.softberry.com/berry.phtml?topic=bprom&group=programs&subgroup=gfindb.

For manuscripts utilizing custom algorithms or software that are central to the research but not yet described in published literature, software must be made available to editors and reviewers. We strongly encourage code deposition in a community repository (e.g. GitHub). See the Nature Portfolio guidelines for submitting code & software for further information.

Data

Policy information about availability of data

All manuscripts must include a data availability statement. This statement should provide the following information, where applicable:

- Accession codes, unique identifiers, or web links for publicly available datasets
- A description of any restrictions on data availability
- For clinical datasets or third party data, please ensure that the statement adheres to our policy

Whole genome sequencing data that support the findings of this study have been deposited in the NCBI SRA database with the accession number PRJNA783062 [https://www.ncbi.nlm.nih.gov/sra/?term=PRJNA783062]. PP_2569 can be accessed in Uniprot database using the link [https://www.uniprot.org/uniprotkb/Q88JS8/

entry]. Source data are provided with this paper.

Field-specific reporting

Please select the one below that is the best fit for your research. If you are not sure, read the appropriate sections before making your selection.

- [x] Life sciences
- [ ] Behavioural & social sciences
- [ ] Ecological, evolutionary & environmental sciences

For a reference copy of the document with all sections, see nature.com/documents/nr-reporting-summary-flat.pdf

Life sciences study design

All studies must disclose on these points even when the disclosure is negative.

Sample size	The sample size (N=3) of shake flasks and plate reader experiments, and the sample size (N=1 or 2) of bioreactor cultivation were determined by experience in this research area, which has been proven consistent and sufficient to support the conclusions in the research area.
Data exclusions	No data were excluded.
Replication	Triplicates were used for all the shake flask and plate reader experiments, strains QP328, QP478, and LC224 have been evaluated in plate reader and shake flasks for several times, and all attempts at replication were successful. Duplicates and singlets were used for bioreactor cultivations. Several other batches of bioreactor cultivations of the strain LC224 have been repeated for future work, and all the results are reproducible.
Randomization	Bacterial cultures were randomly assigned to different strains. Shake flasks of different strains were randomly distributed in the incubator.
Blinding	Investigators performing the experiment were blinded to data collection. The researchers performing analytics were blinded for strain information.

Reporting for specific materials, systems and methods

We require information from authors about some types of materials, experimental systems and methods used in many studies. Here, indicate whether each material, system or method listed is relevant to your study. If you are not sure if a list item applies to your research, read the appropriate section before selecting a response.

Materials & experimental systems

n/a	Involved in the study	
☒	☐	Antibodies
☒	☐	Eukaryotic cell lines
☒	☐	Palaeontology and archaeology
☒	☐	Animals and other organisms
☒	☐	Human research participants
☒	☐	Clinical data
☒	☐	Dual use research of concern

Methods

n/a	Involved in the study	
☒	☐	ChIP-seq
☒	☐	Flow cytometry
☒	☐	MRI-based neuroimaging
